# Sterically Stabilized Diblock Copolymer Nanoparticles Enable Efficient Preparation of Non-Aqueous Pickering Nanoemulsions

**DOI:** 10.1021/acs.langmuir.3c00464

**Published:** 2023-05-15

**Authors:** Saul J. Hunter, Steven P. Armes

**Affiliations:** Department of Chemistry, Brook Hill, University of Sheffield, Dainton Building, Sheffield, South Yorkshire S3 7HF, U.K.

## Abstract

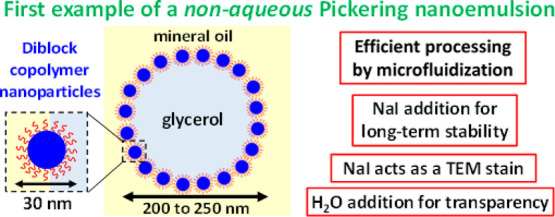

We report the first example of a non-aqueous Pickering nanoemulsion, which comprises glycerol droplets dispersed in mineral oil. The droplet phase is stabilized by hydrophobic sterically stabilized poly(lauryl methacrylate)-poly(benzyl methacrylate) nanoparticles which are prepared directly in mineral oil using polymerization-induced self-assembly. First, a glycerol-in-mineral oil Pickering macroemulsion with a mean droplet diameter of 2.1 ± 0.9 μm is prepared via high-shear homogenization using excess nanoparticles as an emulsifier. Then, this precursor macroemulsion is subjected to high-pressure microfluidization (a single pass at an applied pressure of 20,000 psi) to produce glycerol droplets of approximately 200–250 nm diameter. Transmission electron microscopy studies indicate preservation of the distinctive superstructure produced by nanoparticle adsorption at the glycerol/mineral oil interface, thus confirming the Pickering nature of the nanoemulsion. Glycerol is sparingly soluble in mineral oil, thus such nanoemulsions are rather susceptible to destabilization via Ostwald ripening. Indeed, substantial droplet growth occurs within 24 h at 20 °C, as judged by dynamic light scattering. However, this problem can be suppressed by dissolving a non-volatile solute (sodium iodide) in glycerol prior to formation of the nanoemulsion. This reduces diffusional loss of glycerol molecules from the droplets, with analytical centrifugation studies indicating much better long-term stability for such Pickering nanoemulsions (up to 21 weeks). Finally, the addition of just 5% water to the glycerol phase prior to emulsification enables the refractive index of the droplet phase to be matched to that of the continuous phase, leading to relatively transparent nanoemulsions.

## Introduction

Pickering emulsions comprise liquid droplets (e.g., oil or water) dispersed within a second immiscible liquid (e.g., water or oil) in which the droplets are coated with a layer of adsorbed particles.^1−7^ Adsorption at the liquid–liquid interface occurs spontaneously because it minimizes the interfacial area between the two immiscible liquids, which lowers the free energy of the system.^3,5^ Many types of particles have been used in this context: hydrophilic particles usually lead to the formation of an oil-in-water emulsion, whereas hydrophobic particles favor the formation of water-in-oil emulsions.^3,5,8−12^ Pickering emulsions typically exhibit greater long-term stability than conventional surfactant-stabilized emulsions; they also offer reduced foaming during homogenization and better reproducibility.^3^

Nanoemulsions are relatively fine emulsions that can be prepared by temperature-induced phase inversion,^13,14^ emulsion inversion point,^15,16^ or high-energy emulsification^17−20^ of an existing coarse emulsion. Precisely what constitutes a nanoemulsion seems to be a somewhat controversial topic in the literature.^21^ For example, Anton et al. defined a nanoemulsion to contain droplets of less than 500 nm diameter,^22^ whereas Solans and co-workers suggested a rather stricter characteristic length scale of less than 200 nm diameter.^23^ However, there appears to be general agreement that nanoemulsions tend to exhibit long-term instability owing to Ostwald ripening.^24−29^ In principle, this problem can be suppressed provided that the droplet phase is sufficiently insoluble within the continuous phase.^27,30,31^ There are numerous literature reports of surfactant-stabilized nanoemulsions^23,32−35^ but far fewer examples of Pickering nanoemulsions.^36−46^ This is no doubt because there are only a limited number of examples of colloidal particles that are (i) sufficiently small to act as effective emulsifiers and (ii) possess minimal surface charge (because highly charged particles tend not to adsorb efficiently at the liquid–liquid interface).

Polymerization-induced self-assembly (PISA) has become widely recognized as an efficient and versatile route to a wide range of sterically stabilized diblock copolymer nanoparticles.^47−52^ Importantly, PISA can be conducted in either polar or non-polar solvents, which enables the rational design of either hydrophilic or hydrophobic nanoparticles. Over the past seven years, we have reported that such nanoparticles can be used to stabilize either oil-in-water^53−56^ or water-in-oil^57^ Pickering nanoemulsions, respectively.

**Scheme 1:**
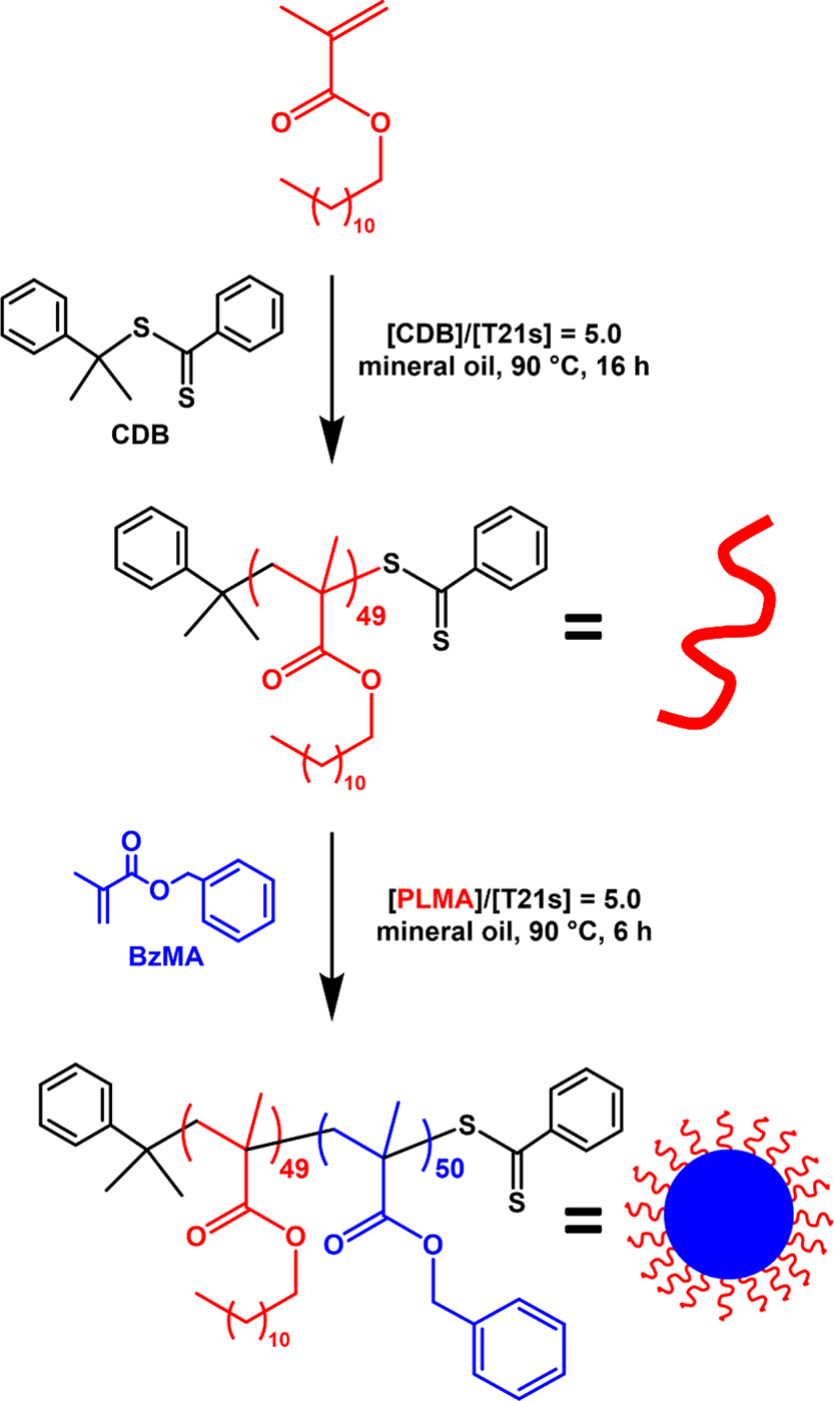
Reaction Scheme for the Synthesis of PLMA_49_-PBzMA_50_ Diblock Copolymer Nanoparticles via RAFT Dispersion Polymerization of BzMA in Mineral Oil at 90 °C

**Scheme 2:**
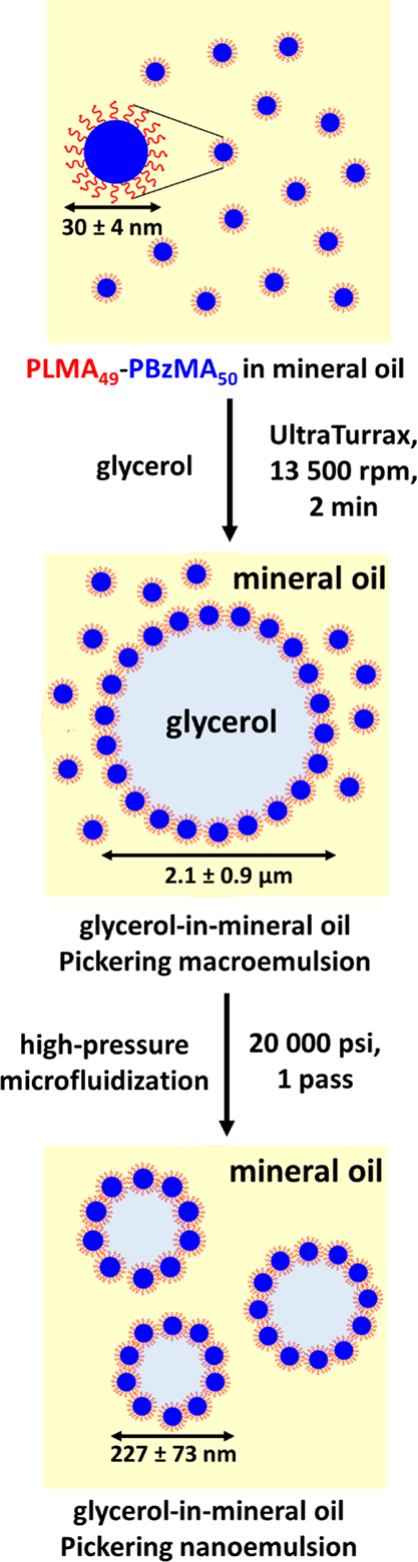
Schematic Representation of the Two-Step Preparation of a Non-Aqueous Pickering Nanoemulsion Using PLMA_49_-PBzMA_50_ Nanoparticles First, a glycerol-in-mineral oil Pickering macroemulsion of 2.1 ± 0.9 μm diameter is prepared via high-shear homogenization (UltraTurrax overhead stirrer, 13,500 rpm for 2 min at 20 °C). This relatively coarse precursor emulsion is then refined by just one pass through a commercial LV1 microfluidizer at 20,000 psi to obtain a glycerol-in-mineral oil Pickering nanoemulsion comprising glycerol droplets of 227 ± 73 nm diameter.

Herein we report the first example of a *non-aqueous* Pickering nanoemulsion. This is achieved by preparing hydrophobic sterically stabilized diblock copolymer nanoparticles of 30 ± 4 nm diameter directly in mineral oil via the reversible addition-fragmentation chain transfer (RAFT) dispersion polymerization of benzyl methacrylate (BzMA) using an oil-soluble poly(lauryl methacrylate) (PLMA) precursor (see Scheme 1). Similar PISA formulations have been previously employed by Armes and co-workers to produce hydrophobic nanoparticles for the preparation of water-in-oil Pickering (nano)emulsions.^11,57−60^ The resulting nanoparticles are used to prepare a relatively coarse Pickering macroemulsion via high-shear homogenization with glycerol, which is immiscible with the mineral oil. Excess nanoparticles are deliberately employed during this initial step, with subsequent high-pressure microfluidization producing the desired nanoemulsion Pickering nanoemulsion, which comprises glycerol droplets of approximately 200–250 nm diameter dispersed within mineral oil (see Scheme 2). The Pickering nature of the droplets is confirmed by transmission electron microscopy (TEM) studies. Finally, the effect of dissolving a suitable non-volatile solute within the droplet phase on the susceptibility of such Pickering nanoemulsions toward Ostwald ripening is assessed using analytical centrifugation.

## Experimental Section

### Materials

Lauryl methacrylate (LMA, 96%), benzyl methacrylate (BzMA, 96%), cumyl dithiobenzoate (CDB), and CDCl_3_ were purchased from Merck (UK). Each monomer was passed through basic alumina in order to remove the inhibitor prior to use. *tert*-Butyl peroxy-2-ethylhexanoate (Trigonox 21S or T21s) initiator was supplied by AkzoNobel (The Netherlands). Glycerol, sodium iodide (NaI), and tetrahydrofuran (THF) were purchased from VWR (UK). Group III hydroisomerized mineral oil (viscosity = 3.1 cSt at 100 °C; refractive index = 1.462) was kindly provided by The Lubrizol Corporation Ltd. (Hazelwood, Derbyshire, UK). Deionized water was used for all experiments.

#### One-Pot Synthesis of PLMA_49_-PBzMA_50_ Diblock Copolymer Nanoparticles in Mineral Oil

The one-pot synthesis of PLMA_49_-PBzMA_50_ diblock copolymer spheres was conducted as follows. LMA (2.333 g; 9.18 mmol), CDB (50.0 mg; 183.5 μmol; target degree of polymerization = 50), and T21s initiator (9.93 mg; 45.88 μmol; dissolved at 10% v/v in mineral oil; [T21s]/[CDB] = 5) were dissolved in mineral oil (3.58 g; target solids = 40% w/w). The reaction mixture was sealed in a 100 mL round-bottomed flask and purged with nitrogen gas for 30 min. The deoxygenated solution was then placed in a pre-heated oil bath at 90 °C for 16 h (final LMA conversion = 97%; *M*_n_ = 13,400 g mol^–1^; *M*_w_/*M*_n_ = 1.13). BzMA (1.620 g; 9.17 mmol; target degree of polymerization = 50) and T21s initiator (9.93 mg; 45.88 μmol; dissolved at 10% v/v in mineral oil; [T21s]/[CDB] = 5) were dissolved in mineral oil (12.43 g; target solids = 20% w/w) and purged with nitrogen gas for 30 min before being added to the original reaction vessel at high (97%) LMA conversion. The final BzMA conversion was 99% after 6 h at 90 °C (*M*_n_ = 20,000 g mol^–1^; *M*_w_/*M*_n_ = 1.19).

#### Preparation of Non-Aqueous Pickering Macroemulsions Using High-Shear Homogenization

A 5.0% w/w dispersion of PLMA_49_-PBzMA_50_ diblock copolymer nanoparticles in mineral oil (4.50 mL) was added to a 14 mL glass vial. This was then homogenized with a series of glycerol solutions (0.50 mL; volume fraction = 0.20; containing 0–4.00 M NaI) for 2 min at 20 °C using an IKA Ultra-Turrax T-18 homogenizer equipped with a 10 mm dispersing tool and operating at 13500 rpm.

#### Preparation of Non-Aqueous Pickering Nanoemulsions Using High-Pressure Microfluidization

To study the effect of varying the copolymer concentration on the mean droplet diameter, the initial Pickering macroemulsions (5.0 mL; volume fraction = 0.20; nanoparticle concentration in mineral oil = 1.0 to 7.0% w/w) were further processed using the LV1 microfluidizer. The pressure was fixed at 20,000 psi, and each macroemulsion was passed just once through the LV1 unit to produce the corresponding glycerol-in-mineral oil Pickering nanoemulsion.

To study the effect of varying the applied pressure on the mean droplet diameter, multiple identical batches of a Pickering macroemulsion (5.0 mL; volume fraction = 0.20; nanoparticle concentration in mineral oil = 5.0% w/w) were further processed using the same LV1 microfluidizer. The pressure was systematically varied between 5,000 and 30,000 psi, and each macroemulsion was passed just once through the LV1 unit to produce the corresponding glycerol-in-mineral oil Pickering nanoemulsion.

The optimized protocol for the preparation of non-aqueous Pickering nanoemulsions was as follows. A Pickering macroemulsion (5.0 mL; volume fraction = 0.20; initial nanoparticle concentration in mineral oil = 5.0% w/w) was processed using an LV1 microfluidizer (Microfluidics, USA) at a fixed pressure of 20,000 psi, and each emulsion was passed just once through the LV1 unit to produce the corresponding glycerol-in-mineral oil Pickering nanoemulsion.

To study the effect of varying the concentration of NaI on the stability of the final Pickering nanoemulsions, different batches of a Pickering macroemulsion (5.0 mL; volume fraction = 0.20; NaI concentration = 0.25–4.00 M; nanoparticle concentration in mineral oil = 5.0% w/w) were further processed using the LV1 microfluidizer (Microfluidics, USA) at a fixed pressure of 20,000 psi, and each emulsion was passed just once through the LV1 unit to produce the corresponding glycerol-in-mineral oil Pickering nanoemulsion.

#### Preparation of Transparent Pickering Nanoemulsions Using High-Pressure Microfluidization

A 5.0% w/w dispersion of PLMA_49_-PBzMA_50_ diblock copolymer nanoparticles in mineral oil (4.50 mL) was added to a 14 mL glass vial. This was then homogenized with various aqueous /glycerol solutions (0.50 mL; volume fraction = 0.20; 3–6% v/v water; prepared using deionized water at around pH 6) for 2 min at 20 °C using an IKA Ultra-Turrax T-18 homogenizer equipped with a 10 mm dispersing tool and operating at 13,500 rpm. Each Pickering macroemulsion (5.0 mL, initial nanoparticle concentration in the mineral oil phase = 5.0% w/w) was further processed using the LV1 microfluidizer.

### Characterization

#### ^1^H NMR Spectroscopy

^1^H NMR spectra were recorded in CDCl_3_ using a 400 MHz Bruker Avance spectrometer at 25 °C. Typically 64 scans were averaged per spectrum.

#### Gel Permeation Chromatography (GPC)

Molecular weight distributions were assessed by GPC using THF eluent. The GPC system was equipped with two 5 μm (30 cm) Mixed C columns and a WellChrom K-2301 refractive index detector operating at 950 ± 30 nm. The THF mobile phase contained 2.0% v/v triethylamine and 0.05% w/v butylhydroxytoluene (BHT), and the flow rate was fixed at 1.0 mL min^–1^. A series of twelve near-monodisperse poly(methyl methacrylate) calibration standards (*M*_p_ values ranging from 800 to 2,200,000 g mol^–1^) were used in combination with the refractive index detector.

#### Transmission Electron Microscopy

The staining agent was prepared by dissolving ruthenium(IV) oxide hydrate (0.30 g) and sodium periodate (2.00 g) in water (50 mL). Nanoparticle dispersions in mineral oil were diluted to 0.02% w/w using *n*-dodecane. A droplet (10 μL) was then placed on a carbon-coated copper TEM grid with the aid of a micropipet. Each grid was then stained for 7 min by exposure to the heavy metal stain within a desiccator. Glycerol-in-mineral oil nanoemulsions containing various amounts of NaI within the glycerol droplets were diluted to 1.0% v/v using *n*-dodecane, placed on a carbon-coated copper TEM grid with the aid of a micropipet, and viewed without using any heavy metal stain. TEM images were recorded using a Tecnai Spirit T12 TEM instrument operating at 80 kV and equipped with an Orius SC1000B S4 CCD camera (2672 × 4008 pixels; 9 μm each).

#### Dynamic Light Scattering (DLS)

Hydrodynamic *z*-average diameters were obtained by using a Malvern Zetasizer NanoZS instrument at a fixed scattering angle of 173°. 0.1% w/w nanoemulsions or nanoparticle dispersions were analyzed using disposable cuvettes and the results were averaged over three consecutive runs, each comprising ten analyses. The mineral oil used to dilute each sample was ultrafiltered through a 0.20 μm membrane to remove extraneous dust.

#### Analytical Centrifugation

Droplet size distributions were assessed using a LUMiSizer analytical photocentrifuge (LUM GmbH, Berlin, Germany) at 20 °C. Measurements were conducted on dilute Pickering nanoemulsions (1.0% v/v glycerol) using 2 mm pathlength polyamide cells at 500 rpm for 200 profiles (allowing 10 s between profiles), and the rate of centrifugation was subsequently increased up to 4000 rpm for a further 800 profiles. The slow initial rate of centrifugation enabled detection of any larger oil droplets that might be present within the nanoemulsion. Overall, the measurement time was approximately 135 min. The LUMiSizer instrument employs space- and time-resolved extinction profiles (STEP) technology to measure the intensity of transmitted near-infrared light as a function of time and position simultaneously over the entire length of the cell. The gradual progression of these transmission profiles over time provides information on the rate of sedimentation of the glycerol droplets and hence enables assessment of the droplet size distribution. The particle density is an essential input parameter for analytical centrifugation studies. The droplet density used for the nanoemulsion aging studies was either the density of pure glycerol or the appropriate density for a given glycerol/NaI solution (which are 1.33, 1.47, 1.60, 1.88, and 2.44 g cm^–3^ for 0.25, 0.50, 1.00, 2.00, and 4.00 M NaI, respectively).^61^ This ignores any contribution to the droplet density from the adsorbed PLMA_49_-PBzMA_50_ nanoparticles. This is a reasonable approximation given that we merely wish to assess *relative* changes in the droplet size distribution over time.

#### Visible Absorption Spectroscopy

The transmittance of (glycerol plus water)-in-mineral oil Pickering nanoemulsions (volume fraction of glycerol plus water = 0.20) was studied using a PC-controlled UV-1800 spectrophotometer equipped with a 10 mm pathlength quartz cell. Spectra were recorded between 200 and 800 nm at 20 °C.

#### Small-Angle X-ray Scattering (SAXS)

SAXS patterns were recorded at a synchrotron facility (ESRF, beamline ID02, Grenoble, France)^62^ using a monochromatic X-ray source (wavelength λ = 0.0995 nm, with *q* ranging from 0.0021 to 2.0 nm^–1^, where *q* is the length of the scattering vector, i.e., *q* = (4π/λ) sin θ, and θ is the one-half of the scattering angle) and a Ravonix MX-170HS CCD detector. A flow-through glass capillary (2.0 mm diameter) was connected to an injector syringe and a waste container via plastic tubing and mounted horizontally on the beamline stage; this setup was used as a sample holder. Scattering data were reduced using standard routines from the beamline^62^ and were further analyzed using Irena SAS macros^63^ for Igor Pro.

## Results and Discussion

The nanoparticles used in the current study were prepared by chain-extending an oil-soluble PLMA precursor with BzMA in mineral oil using a convenient one-pot protocol developed by Derry and co-workers.^64^ Mineral oil was selected as the solvent for this polymerization owing to its greater immiscibility with glycerol than *n*-dodecane. Initially, LMA was polymerized at 70% w/w solids in mineral oil with more than 97% conversion being achieved within 16 h at 90 °C. The resulting PLMA_49_ precursor was subsequently chain-extended with BzMA targeting a mean degree of polymerization of 50 at 20% w/w solids. ^1^H NMR spectroscopy studies indicated that the BzMA polymerization proceeded to 99% conversion within 6 h at 90 °C (see Figure S1). THF GPC analysis indicated a relatively narrow molecular weight distribution for the final copolymer (*M*_n_ = 20,000; *M*_w_/*M*_n_ = 1.19; vs a series of near-monodisperse poly(methyl methacrylate) calibration standards) and a high blocking efficiency, suggesting that this RAFT dispersion polymerization was well-controlled (see Figure S2).^65^

**Figure 1:**
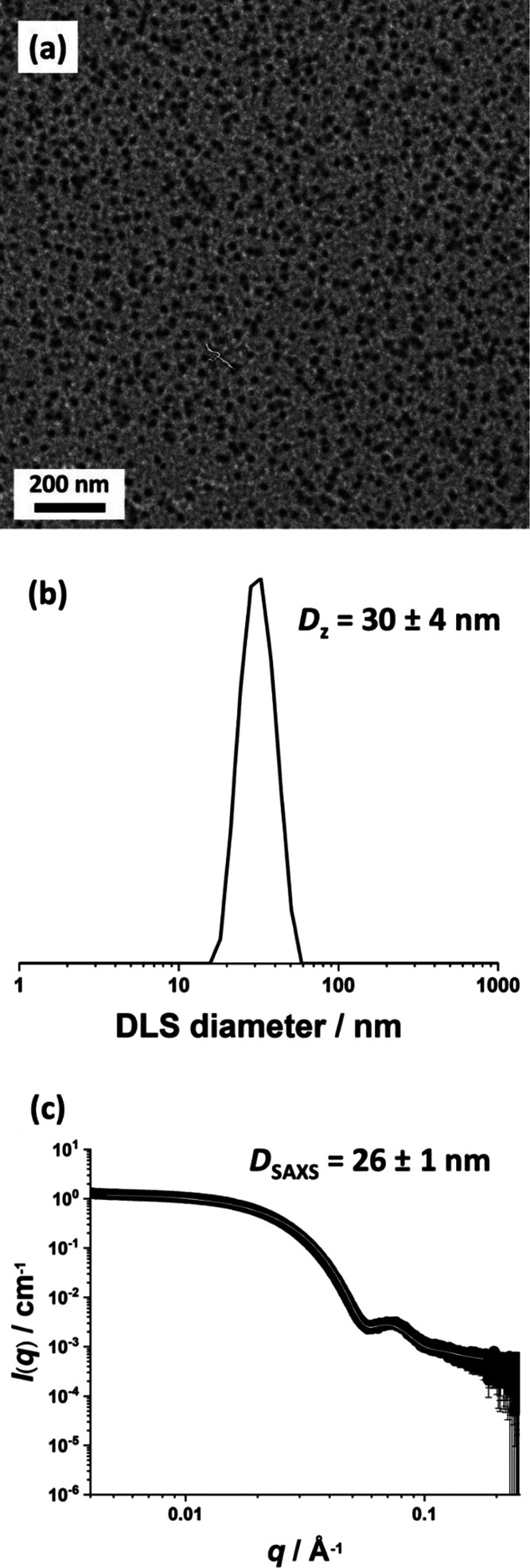
Characterization data obtained for the spherical PLMA_49_-PBzMA_50_ diblock copolymer nanoparticles prepared in mineral oil: (a) (TEM image, (b) DLS data, and (c) SAXS data.

TEM analysis indicated a well-defined spherical morphology for the final PLMA_49_−PBzMA_50_ nanoparticles (Figure 1a). DLS studies reported a hydrodynamic *z*-average diameter of 30 ± 4 nm for these nanoparticles, see Figure 1b. This is consistent with the number-average diameter of 19 ± 3 nm estimated from TEM studies (based on digital image analysis of more than 150 nanoparticles). Fitting the SAXS pattern recorded for these sterically stabilized nanoparticles using a spherical micelle model indicated an overall volume-average diameter of 26 ± 1 nm (Figure 1c). Based on our prior studies, such spherical nanoparticles should be sufficiently small to enable the preparation of glycerol-in-mineral oil Pickering nanoemulsions.^37,53^

Initially, a relatively coarse Pickering macroemulsion with a mean number-average droplet diameter of 2.1 ± 0.9 μm (see Figure S3) was prepared by subjecting a 5.0% w/w dispersion of PLMA_49_-PBzMA_50_ nanoparticles in mineral oil to high-shear homogenization in the presence of glycerol (glycerol volume fraction = 0.20). These conditions were deliberately selected because they lead to a large excess of free nanoparticles. These non-adsorbed nanoparticles are required to stabilize the substantial increase in interfacial area for the glycerol droplets that is generated during the subsequent high-pressure microfluidization to produce the much finer Pickering nanoemulsion. More specifically, the precursor macroemulsion was subjected to just one pass through an LV1 microfluidizer at an applied pressure of 20,000 psi to generate a glycerol-in-mineral oil Pickering nanoemulsion with a mean droplet diameter of 227 ± 73 nm, as illustrated in Scheme 2.

In our prior studies, we reported that the preparation of either oil-in-water or water-in-oil Pickering nanoemulsions required multiple passes through an LV1 microfluidizer. Each pass reduces the droplet size until a minimum diameter is achieved for a given applied pressure. For example, ten passes are required to prepare *n*-dodecane-in-water Pickering nanoemulsions of around 200 nm diameter stabilized by hydrophilic diblock copolymer nanoparticles.^53,54^

In the arguably more pertinent case of water-in-*n*-dodecane Pickering nanoemulsions, five passes through an LV1 microfluidizer were required to produce aqueous droplets of approximately 250 nm diameter.^57^ Clearly, the microfluidization processing conditions required to generate the glycerol-in-mineral oil Pickering nanoemulsion reported in the current study are much less demanding. This important difference can be rationalized by considering the free energy change for emulsification (Δ*G*_emul_), as defined by eq 1:
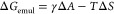
1where Δ*A* is the difference in interfacial area between the initial state (which comprises two immiscible bulk liquids) and the final nanoemulsion and γ is the interfacial tension. The interfacial tension for the *n*-dodecane–water interface is around 53 mN m^–1^, which is significantly greater than that for the *n*-dodecane–glycerol interface (25 mN m^–1^).^66,67^ Thus, significantly less energy is required to generate glycerol droplets in *n*-dodecane (or mineral oil, which has a similar chemical composition) than aqueous droplets in *n*-dodecane. Indeed, using multiple passes to further process the nanoemulsion only led to a rather modest reduction in the mean droplet diameter (see Figure S4). It is also worth emphasizing that fine glycerol droplets can be produced in the absence of any salt, whereas the addition of NaCl to the aqueous droplet phase is essential to generate a water-in-*n*-dodecane nanoemulsion.^57^

**Figure 2:**
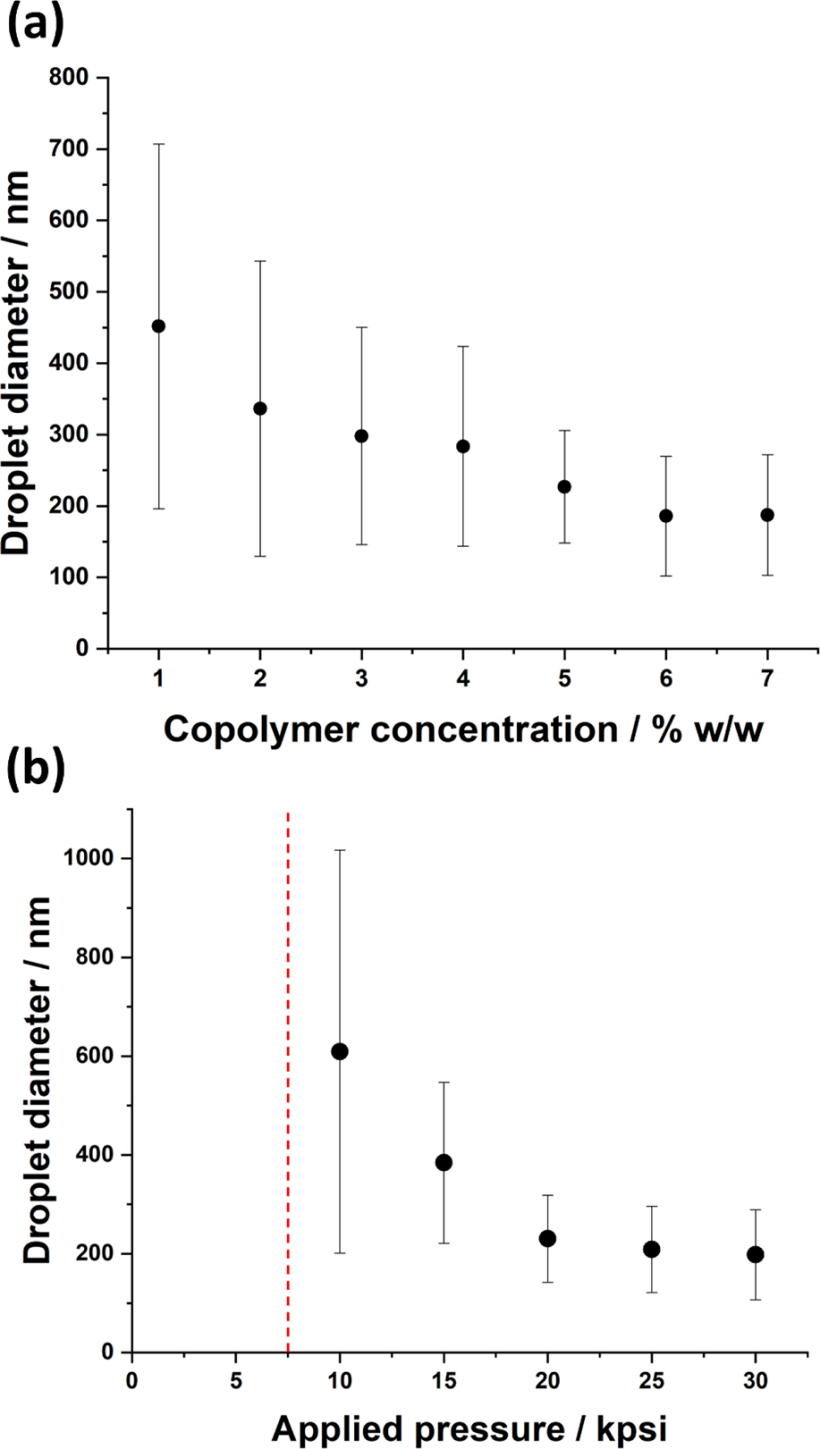
Variation in the *z*-average diameter of glycerol droplets with (a) nanoparticle concentration (at a fixed applied pressure of 20,000 psi) and (b) applied pressure (at a fixed nanoparticle concentration of 5.0% w/w) for glycerol-in-mineral oil Pickering nanoemulsions prepared using PLMA_49_-PBzMA_50_ nanoparticles in the absence of any added NaI. Conditions: glycerol volume fraction = 0.20; one pass through an LV1 microfluidizer. Error bars represent standard deviations for the DLS droplet size distributions rather than the experimental error associated with repeated measurements. The vertical dashed red line shown in part (b) indicates that a nanoemulsion could not be formed at (or below) this pressure.

Thompson et al. reported that the mean droplet diameter of oil-in-water Pickering nanoemulsions could be tuned by systematically varying the copolymer concentration and applied pressure employed during microfluidization. To examine whether this was also the case for glycerol-in-mineral oil nanoemulsions, the PLMA_49_-PBzMA_50_ nanoparticle concentration was varied from 1 to 7% w/w at a constant applied pressure of 20,000 psi (see Figure 2a). As expected, increasing the nanoparticle concentration up to 6% w/w led to a gradual (albeit modest) reduction in the *z*-average droplet diameter reported by DLS. Using a higher nanoparticle concentration aids the formation of smaller glycerol droplets since there are more nanoparticles available to stabilize the additional interfacial area generated during microfluidization. Similarly, raising the applied pressure from 10,000 to 20,000 psi at a fixed nanoparticle concentration of 5.0% w/w leads to a reduction in the mean droplet diameter (see Figure 2b). This is because finer droplets can be generated at higher pressures. However, increasing the applied pressure above 20,000 psi led to no significant further reduction in mean droplet diameter.

**Figure 3:**
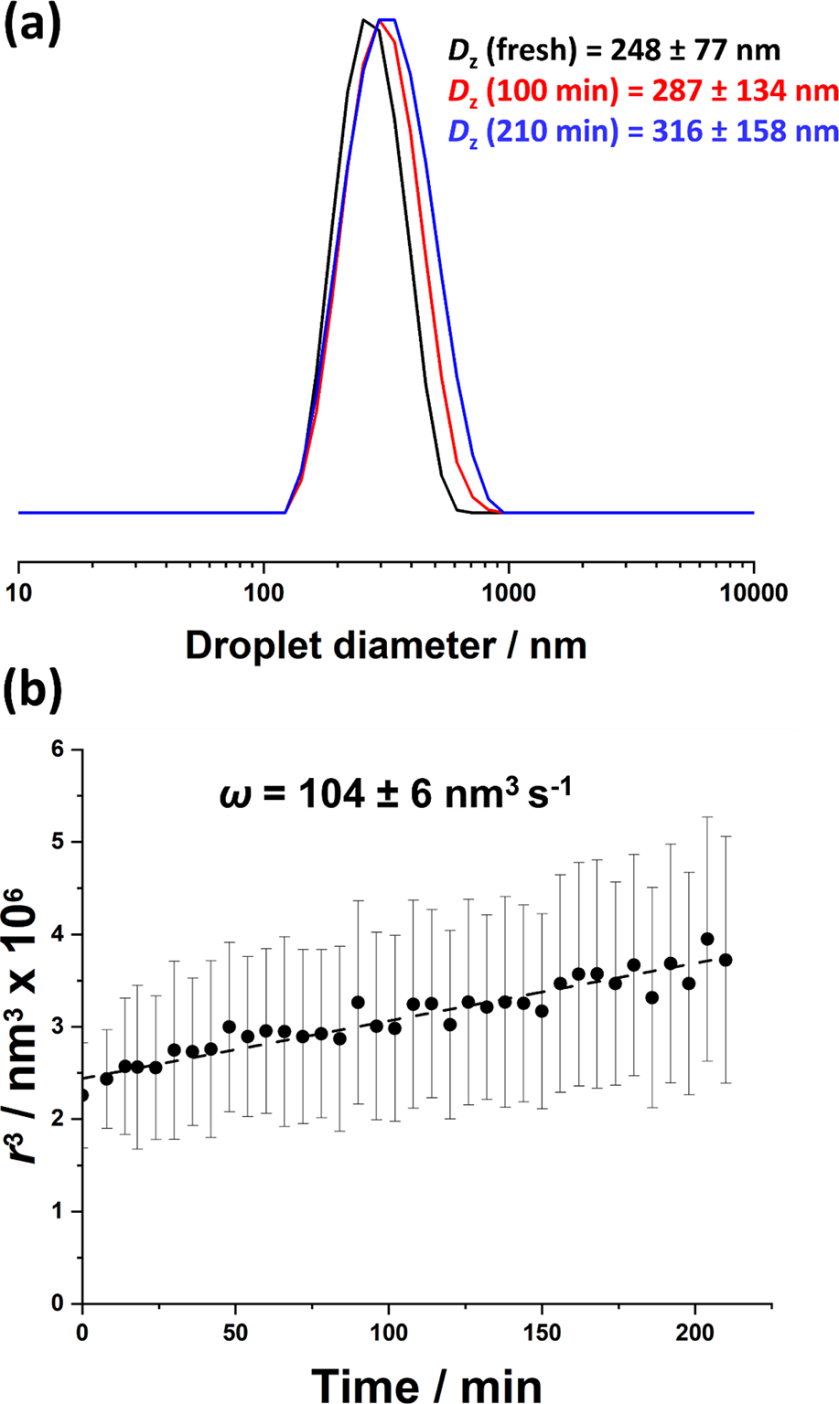
(a) DLS traces recorded for a glycerol-in-mineral oil Pickering nanoemulsion: freshly made (black curve), aged for 100 min (red curve), and aged for 210 min (blue curve). (b) Variation in the cube of the mean droplet radius (*r*^3^) at 20 °C, as determined by DLS studies of aging glycerol-in-mineral oil Pickering nanoemulsions prepared using 5.0% w/w PLMA_49_-PBzMA_50_ nanoparticles. Conditions: applied pressure = 20,000 psi; 1 pass; glycerol volume fraction = 0.20.

**Figure 4:**
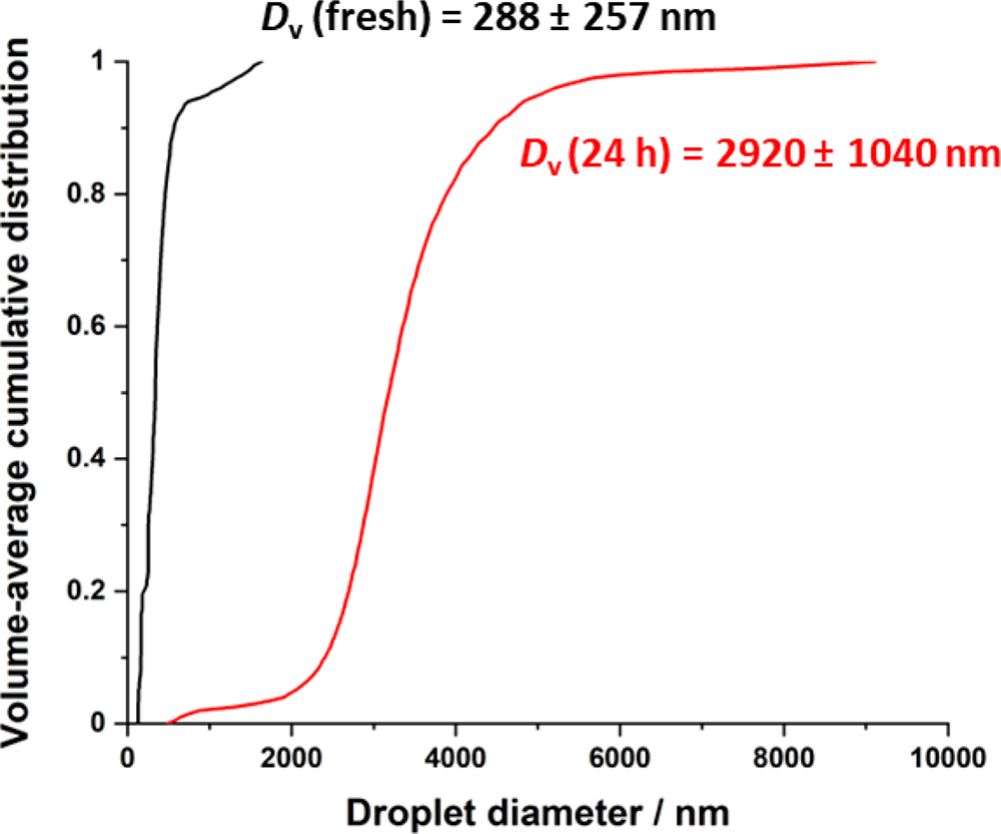
Analytical centrifugation (LUMiSizer instrument) traces recorded for freshly made and one-day-old glycerol-in-mineral oil Pickering nanoemulsions. Conditions: applied pressure = 20,000 psi; 1 pass; glycerol volume fraction = 0.20.

Moreover, our prior studies suggested that nanoparticle disintegration can occur at higher applied pressures, leading to the generation of individual diblock copolymer chains.^53,56^ Thus such conditions are best avoided if genuine Pickering nanoemulsions are desired. In view of these preliminary observations, a nanoparticle concentration of 5% w/w and a single pass at an applied pressure of 20,000 psi were used for the remaining experiments. Unfortunately, visual inspection of a glycerol-in-mineral oil Pickering nanoemulsion prepared using the above optimized conditions confirmed that demulsification occurred within 24 h (see Figure S5). Nanoemulsions are known to be susceptible to Ostwald ripening,^23^ so such instability is likely to be related to the background solubility of glycerol within the mineral oil continuous phase. To examine this hypothesis, the *z*-average diameter of a freshly prepared nanoemulsion was recorded every 6 min using DLS. The observed variation in the cube of the mean droplet radius (*r*^3^) over time is shown in Figure 3. An approximately linear relationship is observed over the first 210 min. According to Lifshitz−Slyozov−Wagner (LSW) theory,^68,69^ this indicates that droplet growth occurs predominantly via Ostwald ripening, which is driven by the relatively high Laplace pressure within such small droplets.^37,54^ Furthermore, the rate of Ostwald ripening was calculated to be 104 ± 6 nm^3^ s^–1^ from the gradient of this linear plot. Clearly, the glycerol droplets undergo substantial ripening in mineral oil within relatively short time scales. The same nanoemulsion was analyzed using DLS and analytical centrifugation. DLS reported a *z*-average diameter of 248 ± 77 nm for the freshly prepared nanoemulsion (see Figure 3a), whereas analytical centrifugation reported a volume-average droplet diameter of 288 ± 257 nm (see Figure 4). This discrepancy most likely arises because the latter measurement requires a significantly longer analysis time (nearly 3 h). According to the data shown in Figure 3, this time period is sufficient for droplet growth to occur via Ostwald ripening. After aging the same Pickering nanoemulsion for 24 h at 20 °C (see Figure 4), its volume-average droplet diameter had increased up to nearly 3 μm, which indicates very poor long-term stability.

It is well-known that the addition of electrolyte to the aqueous phase of surfactant-stabilized nanoemulsions can significantly suppress the rate of Ostwald ripening.^18,70,71^ For example, Koroleva and Yurtov studied the effect of varying the NaCl concentration within the aqueous phase of water-in-mineral oil emulsions.^70^ Nanoemulsions prepared using less than 0.188 M NaCl were unstable with respect to Ostwald ripening, resulting in larger droplets that became susceptible to coalescence. Moreover, Hunter et al. reported that the addition of salt (0.11 M NaCl) was essential to generate relatively stable water-in-*n*-dodecane Pickering nanoemulsions prepared using diblock copolymer nanoparticles.^57^ In principle, the addition of salt to the glycerol droplet phase should also reduce the rate of Ostwald ripening for the glycerol-in-mineral oil Pickering nanoemulsions examined in the current study. This is because diffusion of glycerol molecules into the continuous phase leads to an increase in the salt concentration within the remaining droplets, which is energetically unfavorable. Unfortunately, NaCl has relatively low solubility in glycerol. However, NaI is highly soluble in glycerol, so this alternative salt was selected as a non-volatile solute to suppress Ostwald ripening.

Since the iodide anion has a relatively high electron density, employing a relatively high concentration of NaI should enable TEM visualization of the dried droplets without using a heavy metal stain. To examine this hypothesis, the NaI concentration within the glycerol droplets of the nanoemulsions was systematically increased from 0.25 to 4.00 M. Visual inspection suggested the formation of a Pickering nanoemulsion in each case (see Figure S6). DLS studies indicated that such salt addition increased the mean droplet diameter by less than 10% (see Figure S7). Using a core–shell model reported by Balmer et al.,^72^ the mean number of nanoparticles adsorbed per glycerol droplet, *N*, is calculated to lie between 267 and 376 for a series of six freshly prepared Pickering nanoemulsions with comparable DLS droplet diameters (Table S1). Hence the mean packing efficiency, *P*, for the adsorbed nanoparticles surrounding each glycerol droplet is approximately 29–33%.

**Figure 5:**
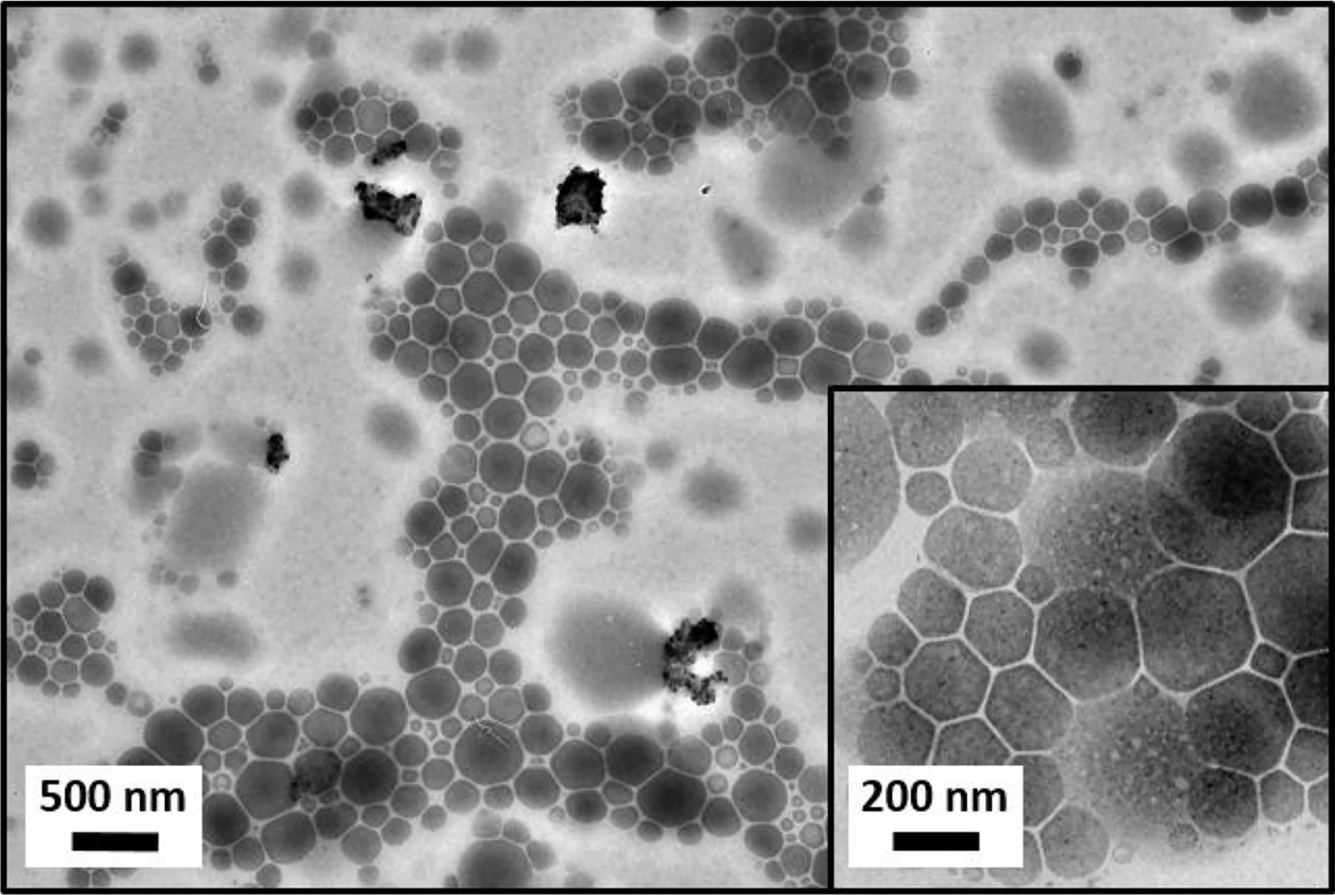
Representative TEM image recorded for a dried glycerol-in-mineral oil nanoemulsion prepared using 5% w/w PLMA_49_-PBzMA_50_ spherical nanoparticles. Conditions: applied pressure = 20,000 psi; 1 pass; NaI concentration = 4.00 M; volume fraction of glycerol + NaI = 0.20.

TEM studies confirmed that the dried droplets became more discernible when employing higher NaI concentrations (see Figure S8). Figure 5 shows a representative TEM image obtained after drying a glycerol-in-mineral oil Pickering nanoemulsion prepared using 4.00 M NaI. Under the ultra-high-vacuum conditions required for TEM, all traces of the glycerol droplets and the mineral oil continuous phase are removed, but the NaI salt remains on the TEM grid. Thus the salt residues provide useful information regarding the original droplet size distribution. Moreover, close inspection indicates that the spherical nanoparticles remain intact during the high-pressure microfluidization conditions, which confirms the Pickering nature of these nanoemulsions (see inset). Analytical centrifugation was used to determine the volume-average size distribution for both fresh and aged Pickering nanoemulsions prepared using various NaI concentrations. We have shown that analytical centrifugation is well-suited for determining the long-term stability of both oil-in-water and water-in-oil Pickering nanoemulsions.^54−57^ Unlike DLS, analytical centrifugation subjects the nanoemulsions to droplet fractionation prior to detection and therefore offers higher resolution.

**Figure 6:**
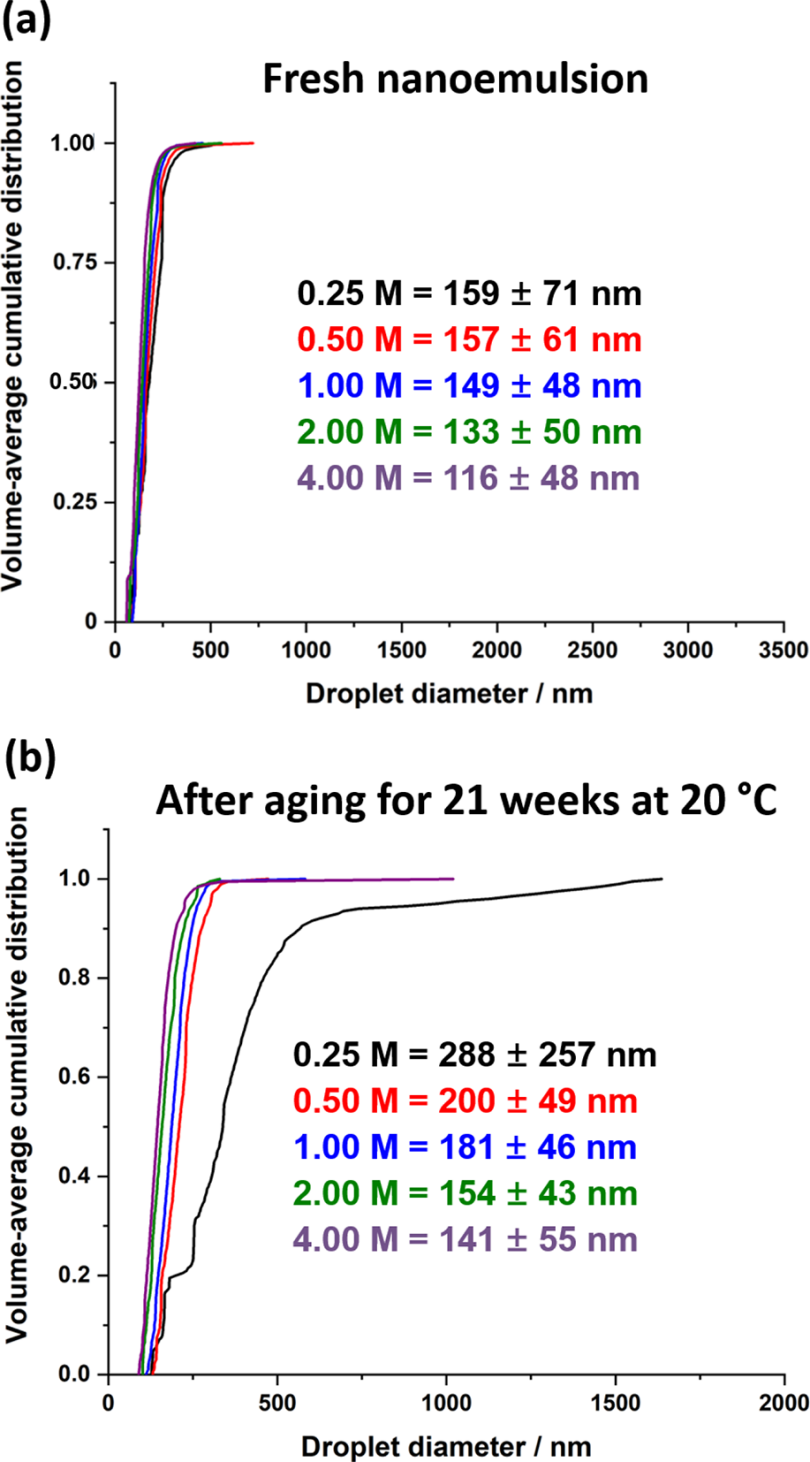
Volume-weighted cumulative size distributions determined by analytical centrifugation (LUMiSizer instrument) for glycerol-in-mineral oil Pickering nanoemulsions prepared using various amounts of NaI dissolved within the glycerol droplet phase: (a) fresh nanoemulsion and (b) after aging for 21 weeks at 20 °C. Microfluidization conditions: applied pressure = 20,000 psi; 1 pass; volume fraction of glycerol/NaI droplet phase = 0.20.

Figure 6 shows the cumulative volume-average distributions recorded for five freshly prepared Pickering nanoemulsions prepared using 0.25, 0.50, 1.00, 2.00, or 4.00 M NaI.

The volume-average diameters for the fresh nanoemulsions are significantly smaller than those reported by DLS. This is mainly because DLS reports the *z*-average diameter, which is more biased toward the larger droplets. There is also some uncertainty regarding the effective density of the glycerol droplets, as well as the likelihood of a density distribution being superimposed on the droplet size distribution.^54,73^ Nevertheless, such analytical centrifugation studies enable the *relative* change in droplet distribution to be monitored over time. After aging for 21 weeks at 20 °C, the nanoemulsion prepared using 0.25 M NaI exhibited a significant increase in its droplet diameter. In contrast, the other four nanoemulsions remained relatively unchanged (only relatively modest increases in droplet diameter are observed). Clearly, dissolution of at least 0.50 M NaI within the glycerol phase prior to high-shear homogenization is required to suppress Ostwald ripening for such non-aqueous Pickering nanoemulsions.

**Figure 7:**
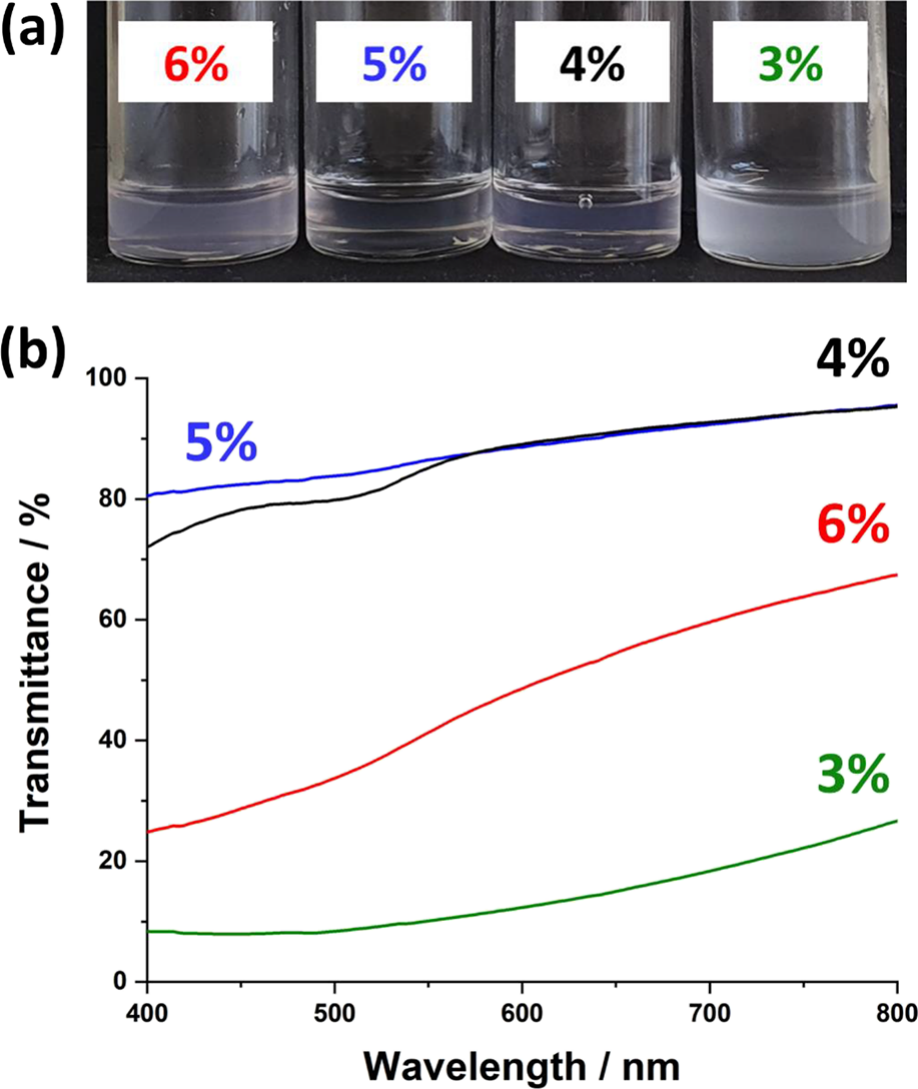
(a) Digital photographs and (b) transmittance vs wavelength plots recorded for 20% v/v glycerol-in-mineral oil Pickering nanoemulsions with 3, 4, 5, or 6% v/v water added to the glycerol droplet phase prior to emulsification. The relatively low refractive index of the water (1.33) enables the refractive index of the droplets to be matched to that of the mineral oil (1.462) to produce a relatively transparent Pickering nanoemulsion. Microfluidization conditions: applied pressure = 20,000 psi; 1 pass.

Finally, we note that the refractive index of pure glycerol (1.474) at 20 °C^74^ is only slightly higher than that of the mineral oil used in this work (1.462). Thus, addition of a relatively small amount of water (refractive index = 1.333)^75^ to glycerol prior to emulsification should be sufficient to lower the refractive index of the droplet phase to match that of the continuous phase. If light scattering from the adsorbed nanoparticles surrounding the droplets can be neglected, this should result in a relatively transparent Pickering nanoemulsion. This hypothesis is explored in Figure 7, which confirms that the addition of just 5% water to glycerol enables the preparation of a relatively transparent nanoemulsion that exhibits 81% (λ = 400 nm) to 96% (λ = 800 nm) transmittance across the visible spectrum even at a droplet volume fraction of 0.20. On the other hand, further addition of water (6%) lowers the refractive index of the droplets *below* that of the mineral oil, which leads to additional light scattering and hence a reduction in transmittance. In principle, even higher transmittance (particularly at shorter wavelengths) should be achievable if the PBzMA-based nanoparticle cores (refractive index ∼1.57)^76^ were replaced by poly(methyl methacrylate) cores (refractive index ∼1.49).^76^ Moreover, such PLMA-PMMA nanoparticles are readily accessible via PISA.^77^ On the other hand, a larger volume of water would be required to produce a *relatively stable* transparent nanoemulsion. This is because the addition of NaI to glycerol raises the refractive index of the droplet phase (see Figure S4).

## Conclusions

We report the first example of a non-aqueous Pickering nanoemulsion using glycerol as the droplet phase and mineral oil as the continuous phase. The glycerol droplets are stabilized using sterically stabilized diblock copolymer nanoparticles of 30 nm diameter that are prepared directly in the mineral oil via PISA. Nanoemulsions are produced from precursor Pickering macroemulsions via high-pressure microfluidization. Notably, only a single pass through the microfluidizer is required to produce glycerol droplets of around 200–250 nm diameter. Such mild processing conditions compare favorably with those required for the analogous water-in-oil Pickering nanoemulsions, for which 7–10 passes through the microfluidizer are typically required. Unfortunately, such Pickering nanoemulsions are susceptible to Ostwald ripening within relatively short time scales (hours) owing to the background solubility of glycerol in mineral oil. However, analytical centrifugation studies confirm that this problem can be substantially suppressed by dissolution of NaI within the glycerol phase prior to initial homogenization. Finally, addition of a small amount of water to the glycerol phase lowers the refractive index of the droplets to that of the mineral oil, which enables the preparation of relatively transparent Pickering nanoemulsions.

## Supporting Information Available

^1^H NMR spectra and GPC data for the PLMA_49_ precursor and PLMA_49_-PBzMA_50_ diblock copolymer; optical microscopy image for the glycerol-in-mineral oil Pickering macroemulsion; variation in *z*-average droplet diameter vs number of passes through the LV1 unit for a glycerol-in-mineral oil Pickering nanoemulsion; digital photographs recorded for a freshly prepared glycerol-in-mineral oil Pickering nanoemulsion and after aging for 24 h at 20 °C; digital photographs and DLS data recorded for a series of glycerol-in-mineral oil Pickering nanoemulsions with 0.25–4.00 M NaI dissolved in the glycerol phase; representative TEM images recorded for dried glycerol-in-mineral oil nanoemulsions containing various amounts of NaI dissolved in the droplet phase; and details of the spherical micelle model used for SAXS analysis (PDF)

## References

[ref1] Pickering S. U. CXCVI.—Emulsions. J. Chem. Soc., Trans. 1907, 91, 2001–2021. 10.1039/CT9079102001.

[ref2] Ramsden W. Separation of Solids in the Surface-Layers of Solutions and ’Suspensions’ (Observations on Surface-Membranes, Bubbles, Emulsions, and Mechanical Coagulation). -- Preliminary Account. Proc. R. Soc. London 1904, 72, 156–164. 10.1098/rspl.1903.0034.

[ref3] Binks B. P. Particles as surfactants—similarities and differences. Curr. Opin. Colloid Interface Sci. 2002, 7, 21–41. 10.1016/S1359-0294(02)00008-0.

[ref4] Binks P. B.; Lumsdon S. O. Stability of oil-in-water emulsions stabilised by silica particles. Phys. Chem. Chem. Phys. 1999, 1, 3007–3016. 10.1039/a902209k.

[ref5] Binks B. P.; Lumsdon S. O. Influence of Particle Wettability on the Type and Stability of Surfactant-Free Emulsions. Langmuir 2000, 16, 8622–8631. 10.1021/la000189s.

[ref6] Aveyard R.; Binks B. P.; Clint J. H. Emulsions stabilised solely by colloidal particles. Adv. Colloid Interface Sci. 2003, 100-102, 503–546. 10.1016/S0001-8686(02)00069-6.

[ref7] Hunter S. J.; Armes S. P. Pickering Emulsifiers Based on Block Copolymer Nanoparticles Prepared by Polymerization-Induced Self-Assembly. Langmuir 2020, 36, 15463–15484. 10.1021/acs.langmuir.0c02595. 33325720 PMC7884006

[ref8] Binks B. P.; Lumsdon S. O. Catastrophic Phase Inversion of Water-in-Oil Emulsions Stabilized by Hydrophobic Silica. Langmuir 2000, 16, 2539–2547. 10.1021/la991081j.

[ref9] Binks B. P.; Murakami R.; Armes S. P.; Fujii S. Temperature-Induced Inversion of Nanoparticle-Stabilized Emulsions. Angew. Chem. Int. Ed. 2005, 117, 4873–4876. 10.1002/ange.200501073. 15988782

[ref10] Thompson K. L.; Mable C. J.; Cockram A.; Warren N. J.; Cunningham V. J.; Jones E. R.; Verber R.; Armes S. P. Are block copolymer worms more effective Pickering emulsifiers than block copolymer spheres?. Soft Matter 2014, 10, 8615–8626. 10.1039/C4SM01724B. 25254485

[ref11] Thompson K. L.; Fielding L. A.; Mykhaylyk O. O.; Lane J. A.; Derry M. J.; Armes S. P. Vermicious thermo-responsive Pickering emulsifiers. Chem. Sci. 2015, 6, 4207–4214. 10.1039/C5SC00598A. 29218187 PMC5707463

[ref12] György C.; Hunter S. J.; Girou C.; Derry M. J.; Armes S. P. Synthesis of poly(stearyl methacrylate)-poly(2-hydroxypropyl methacrylate) diblock copolymer nanoparticles via RAFT dispersion polymerization of 2-hydroxypropyl methacrylate in mineral oil. Polym. Chem. 2020, 11, 4579–4590. 10.1039/D0PY00562B.

[ref13] Izquierdo P.; Esquena J.; Tadros T. F.; Dederen C.; Garcia M. J.; Azemar N.; Solans C. Formation and Stability of Nano-Emulsions Prepared Using the Phase Inversion Temperature Method. Langmuir 2002, 18, 26–30. 10.1021/la010808c.

[ref14] Izquierdo P.; Esquena J.; Tadros T. F.; Dederen J. C.; Feng J.; Garcia-Celma M. J.; Azemar N.; Solans C. Phase Behavior and Nano-emulsion Formation by the Phase Inversion Temperature Method. Langmuir 2004, 20, 6594–6598. 10.1021/la049566h. 15274560

[ref15] Forgiarini A.; Esquena J.; González C.; Solans C. Formation of Nano-emulsions by Low-Energy Emulsification Methods at Constant Temperature. Langmuir 2001, 17, 2076–2083. 10.1021/la001362n.

[ref16] Gupta A.; Badruddoza A. Z. M.; Doyle P. S. A General Route for Nanoemulsion Synthesis Using Low-Energy Methods at Constant Temperature. Langmuir 2017, 33, 7118–7123. 10.1021/acs.langmuir.7b01104. 28654749

[ref17] Meleson K.; Graves S.; Mason T. G. Formation of Concentrated Nanoemulsions by Extreme Shear. Soft Mater. 2004, 2, 109–123. 10.1081/SMTS-200056102.

[ref18] Lee L.; Hancocks R.; Noble I.; Norton I. T. Production of water-in-oil nanoemulsions using high pressure homogenisation: A study on droplet break-up. J. Food Eng. 2014, 131, 33–37. 10.1016/j.jfoodeng.2014.01.024.

[ref19] Kumar H.; Kumar V. Ultrasonication assisted formation and stability of water-in-oil nanoemulsions: Optimization and ternary diagram analysis. Ultrason. Sonochem. 2018, 49, 79–88. 10.1016/j.ultsonch.2018.07.022. 30057181

[ref20] Du Z.; Wang C.; Tai X.; Wang G.; Liu X. Optimization and Characterization of Biocompatible Oil-in-Water Nanoemulsion for Pesticide Delivery. ACS Sustainable Chem. Eng. 2016, 4, 983–991. 10.1021/acssuschemeng.5b01058.

[ref21] McClements D. J. Nanoemulsions versus microemulsions: terminology, differences, and similarities. Soft Matter 2012, 8, 1719–1729. 10.1039/C2SM06903B.

[ref22] Anton N.; Benoit J.-P.; Saulnier P. Design and production of nanoparticles formulated from nano-emulsion templates—A review. J. Controlled Release 2008, 128, 185–199. 10.1016/j.jconrel.2008.02.007. 18374443

[ref23] Solans C.; Izquierdo P.; Nolla J.; Azemar N.; Garcia-Celma M. J. Nano-emulsions. Curr. Opin. Colloid Interface Sci. 2005, 10, 102–110. 10.1016/j.cocis.2005.06.004.

[ref24] Kabalnov A. Thermodynamic and theoretical aspects of emulsions and their stability. Curr. Opin. Colloid Interface Sci. 1998, 3, 270–275. 10.1016/S1359-0294(98)80071-X.

[ref25] Taylor P. Ostwald ripening in emulsions. Adv. Colloid Interface Sci. 1998, 75, 107–163. 10.1016/S0001-8686(98)00035-9. 14672850

[ref26] Kabalnov A. Ostwald Ripening and Related Phenomena. J. Dispersion Sci. Technol. 2001, 22, 1–12. 10.1081/DIS-100102675.

[ref27] Wooster T. J.; Golding M.; Sanguansri P. Impact of Oil Type on Nanoemulsion Formation and Ostwald Ripening Stability. Langmuir 2008, 24, 12758–12765. 10.1021/la801685v. 18850732

[ref28] Delmas T.; Piraux H.; Couffin A.-C.; Texier I.; Vinet F.; Poulin P.; Cates M. E.; Bibette J. How To Prepare and Stabilize Very Small Nanoemulsions. Langmuir 2011, 27, 1683–1692. 10.1021/la104221q. 21226496

[ref29] Rodriguez-Lopez G.; O’Neil Williams Y.; Toro-Mendoza J. Individual and Collective Behavior of Emulsion Droplets Undergoing Ostwald Ripening. Langmuir 2019, 35, 5316–5323. 10.1021/acs.langmuir.8b03959. 30844290

[ref30] Kabal’nov A. S.; Pertzov A. V.; Shchukin E. D. Ostwald ripening in two-component disperse phase systems: Application to emulsion stability. Colloids Surf. 1987, 24, 19–32. 10.1016/0166-6622(87)80258-5.

[ref31] Webster A. J.; Cates M. E. Stabilization of Emulsions by Trapped Species. Langmuir 1998, 14, 2068–2079. 10.1021/la9712597.

[ref32] McClements D. J. Edible nanoemulsions: fabrication, properties, and functional performance. Soft Matter 2011, 7, 2297–2316. 10.1039/C0SM00549E.

[ref33] McClements D. J.; Rao J. Food-Grade Nanoemulsions: Formulation, Fabrication, Properties, Performance, Biological Fate, and Potential Toxicity. Crit. Rev. Food Sci. Nutr. 2011, 51, 285–330. 10.1080/10408398.2011.559558. 21432697

[ref34] Gupta A.; Eral H. B.; Hatton T. A.; Doyle P. S. Nanoemulsions: formation, properties and applications. Soft Matter 2016, 12, 2826–2841. 10.1039/C5SM02958A. 26924445

[ref35] Singh Y.; Meher J. G.; Raval K.; Khan F. A.; Chaurasia M.; Jain N. K.; Chourasia M. K. Nanoemulsion: Concepts, development and applications in drug delivery. J. Controlled Release 2017, 252, 28–49. 10.1016/j.jconrel.2017.03.008. 28279798

[ref36] Gupta R.; Rousseau D. Surface-active solid lipid nanoparticles as Pickering stabilizers for oil-in-water emulsions. Food Funct. 2012, 3, 302–311. 10.1039/c2fo10203j. 22237667

[ref37] Persson K. H.; Blute I. A.; Mira I. C.; Gustafsson J. Creation of well-defined particle stabilized oil-in-water nanoemulsions. Colloids Surf., A 2014, 459, 48–57. 10.1016/j.colsurfa.2014.06.034.

[ref38] Sihler S.; Schrade A.; Cao Z.; Ziener U. Inverse Pickering Emulsions with Droplet Sizes below 500 nm. Langmuir 2015, 31, 10392–10401. 10.1021/acs.langmuir.5b02735. 26348090

[ref39] Jiménez Saelices C.; Capron I. Design of Pickering Micro- and Nanoemulsions Based on the Structural Characteristics of Nanocelluloses. Biomacromolecules 2018, 19, 460–469. 10.1021/acs.biomac.7b01564. 29309726

[ref40] Kang D. J.; Bararnia H.; Anand S. Synthesizing Pickering Nanoemulsions by Vapor Condensation. ACS Appl. Mater. Interfaces 2018, 10, 21746–21754. 10.1021/acsami.8b06467. 29846059

[ref41] Du Z.; Li Q.; Li J.; Su E.; Liu X.; Wan Z.; Yang X. Self-Assembled Egg Yolk Peptide Micellar Nanoparticles as a Versatile Emulsifier for Food-Grade Oil-in-Water Pickering Nanoemulsions. J. Agric. Food Chem. 2019, 67, 11728–11740. 10.1021/acs.jafc.9b04595. 31525998

[ref42] Zhao Q.; Jiang L. X.; Lian Z.; Khoshdel E.; Schumm S.; Huang J. B.; Zhang Q. Q. High internal phase water-in-oil emulsions stabilized by food-grade starch. J. Colloid Interface Sci. 2019, 534, 542–548. 10.1016/j.jcis.2018.09.058. 30253355

[ref43] Xiao Z.; Liu Y.; Niu Y.; Kou X. Cyclodextrin supermolecules as excellent stabilizers for Pickering nanoemulsions. Colloids Surf., A 2020, 588, 124367 10.1016/j.colsurfa.2019.124367.

[ref44] Yang Z.; Wang W.; Wang G.; Tai X. Optimization of low-energy Pickering nanoemulsion stabilized with montmorillonite and nonionic surfactants. Colloids Surf., A 2020, 585, 124098 10.1016/j.colsurfa.2019.124098.

[ref45] Nandy M.; Lahiri B. B.; Philip J. Inter-droplet force between magnetically polarizable Pickering oil-in-water nanoemulsions stabilized with γ-Al2O3 nanoparticles: Role of electrostatic and electric dipolar interactions. J. Colloid Interface Sci. 2022, 607, 1671–1686. 10.1016/j.jcis.2021.09.025. 34592554

[ref46] Gauthier G.; Capron I. Pickering nanoemulsions: An overview of manufacturing processes, formulations, and applications. JCIS Open 2021, 4, 100036 10.1016/j.jciso.2021.100036.

[ref47] Charleux B.; Delaittre G.; Rieger J.; D’Agosto F. Polymerization-Induced Self-Assembly: From Soluble Macromolecules to Block Copolymer Nano-Objects in One Step. Macromolecules 2012, 45, 6753–6765. 10.1021/ma300713f.

[ref48] Warren N. J.; Armes S. P. Polymerization-Induced Self-Assembly of Block Copolymer Nano-objects via RAFT Aqueous Dispersion Polymerization. J. Am. Chem. Soc. 2014, 136, 10174–10185. 10.1021/ja502843f. 24968281 PMC4111214

[ref49] Rieger J. Guidelines for the Synthesis of Block Copolymer Particles of Various Morphologies by RAFT Dispersion Polymerization. Macromol. Rapid Commun. 2015, 36, 1458–1471. 10.1002/marc.201500028. 26010064

[ref50] Canning S. L.; Smith G. N.; Armes S. P. A Critical Appraisal of RAFT-Mediated Polymerization-Induced Self-Assembly. Macromolecules 2016, 49, 1985–2001. 10.1021/acs.macromol.5b02602. 27019522 PMC4806311

[ref51] Derry M. J.; Fielding L. A.; Armes S. P. Polymerization-induced self-assembly of block copolymer nanoparticles via RAFT non-aqueous dispersion polymerization. Prog. Polym. Sci. 2016, 52, 1–18. 10.1016/j.progpolymsci.2015.10.002.

[ref52] D’Agosto F.; Rieger J.; Lansalot M. RAFT-Mediated Polymerization-Induced Self-Assembly. Angew. Chem. Int. Ed. 2020, 59, 8368–8392. 10.1002/anie.201911758. 31584738

[ref53] Thompson K. L.; Cinotti N.; Jones E. R.; Mable C. J.; Fowler P. W.; Armes S. P. Bespoke Diblock Copolymer Nanoparticles Enable the Production of Relatively Stable Oil-in-Water Pickering Nanoemulsions. Langmuir 2017, 33, 12616–12623. 10.1021/acs.langmuir.7b02267. 29022716 PMC5677761

[ref54] Thompson K. L.; Derry M. J.; Hatton F. L.; Armes S. P. Long-Term Stability of n-Alkane-in-Water Pickering Nanoemulsions: Effect of Aqueous Solubility of Droplet Phase on Ostwald Ripening. Langmuir 2018, 34, 9289–9297. 10.1021/acs.langmuir.8b01835. 29999324 PMC6085727

[ref55] Hunter S. J.; Penfold N. J. W.; Chan D. H.; Mykhaylyk O. O.; Armes S. P. How Do Charged End-Groups on the Steric Stabilizer Block Influence the Formation and Long-Term Stability of Pickering Nanoemulsions Prepared Using Sterically Stabilized Diblock Copolymer Nanoparticles?. Langmuir 2020, 36, 769–780. 10.1021/acs.langmuir.9b03389. 31899941

[ref56] Hunter S. J.; Armes S. P. Long-Term Stability of Pickering Nanoemulsions Prepared Using Diblock Copolymer Nanoparticles: Effect of Nanoparticle Core Crosslinking, Oil Type, and the Role Played by Excess Copolymers. Langmuir 2022, 38, 8021–8029. 10.1021/acs.langmuir.2c00821. 35737742 PMC9261185

[ref57] Hunter S. J.; Cornel E. J.; Mykhaylyk O. O.; Armes S. P. Effect of Salt on the Formation and Stability of Water-in-Oil Pickering Nanoemulsions Stabilized by Diblock Copolymer Nanoparticles. Langmuir 2020, 36, 15523–15535. 10.1021/acs.langmuir.0c02742. 33332972 PMC7884014

[ref58] Thompson K. L.; Lane J. A.; Derry M. J.; Armes S. P. Non-aqueous Isorefractive Pickering Emulsions. Langmuir 2015, 31, 4373–4376. 10.1021/acs.langmuir.5b00630. 25844544 PMC4577967

[ref59] Thompson K. L.; Mable C. J.; Lane J. A.; Derry M. J.; Fielding L. A.; Armes S. P. Preparation of Pickering Double Emulsions Using Block Copolymer Worms. Langmuir 2015, 31, 4137–4144. 10.1021/acs.langmuir.5b00741. 25834923 PMC4415048

[ref60] Rymaruk M. J.; Thompson K. L.; Derry M. J.; Warren N. J.; Ratcliffe L. P. D.; Williams C. N.; Brown S. L.; Armes S. P. Bespoke contrast-matched diblock copolymer nanoparticles enable the rational design of highly transparent Pickering double emulsions. Nanoscale 2016, 8, 14497–14506. 10.1039/C6NR03856E. 27406976 PMC5047046

[ref61] Gates J. A.; Wood R. H. Densities of aqueous solutions of sodium chloride, magnesium chloride, potassium chloride, sodium bromide, lithium chloride, and calcium chloride from 0.05 to 5.0 mol kg-1 and 0.1013 to 40 MPa at 298.15 K. J. Chem. Eng. Data 1985, 30, 44–49. 10.1021/je00039a015.

[ref62] Narayanan T.; Sztucki M.; Vaerenbergh P.; Léonardon J.; Gorini J.; Claustre L.; Sever F.; Morse J.; Boesecke P. A multipurpose instrument for time-resolved ultra-small-angle and coherent X-ray scattering. J. Appl. Crystallogr. 2018, 51, 1511–1524. 10.1107/S1600576718012748. 30546286 PMC6276275

[ref63] Ilavsky J.; Jemian P. R. Irena: tool suite for modeling and analysis of small-angle scattering. J. Appl. Crystallogr. 2009, 42, 347–353. 10.1107/S0021889809002222.

[ref64] Derry M. J.; Fielding L. A.; Armes S. P. Industrially-relevant polymerization-induced self-assembly formulations in non-polar solvents: RAFT dispersion polymerization of benzyl methacrylate. Polym. Chem. 2015, 6, 3054–3062. 10.1039/C5PY00157A.

[ref65] Chiefari J.; Chong Y. K.; Ercole F.; Krstina J.; Jeffery J.; Le T. P. T.; Mayadunne R. T. A.; Meijs G. F.; Moad C. L.; Moad G.; Rizzardo E.; Thang S. H. Living Free-Radical Polymerization by Reversible Addition–Fragmentation Chain Transfer: The RAFT Process. Macromolecules 1998, 31, 5559–5562. 10.1021/ma9804951.

[ref66] Jańczuk B.; Białopiotrowicz T.; Wójcik W. The components of surface tension of liquids and their usefulness in determinations of surface free energy of solids. J. Colloid Interface Sci. 1989, 127, 59–66. 10.1016/0021-9797(89)90007-6.

[ref67] Jańczuk B.; Wójcik W.; Zdziennicka A. Determination of the Components of the Surface Tension of Some Liquids from Interfacial Liquid-Liquid Tension Measurements. J. Colloid Interface Sci. 1993, 157, 384–393. 10.1006/jcis.1993.1200.

[ref68] Lifshitz I. M.; Slyozov V. V. The kinetics of precipitation from supersaturated solid solutions. J. Phys. Chem. Solids 1961, 19, 35–50. 10.1016/0022-3697(61)90054-3.

[ref69] Wagner C. Theorie der Alterung von Niederschlägen durch Umlösen (Ostwald-Reifung). Z. Elektrochem. Berichte der Bunsengesellschaft Phys. Chem. 1961, 65, 581–591. 10.1002/bbpc.19610650704.

[ref70] Koroleva M. Y.; Yurtov E. V. Effect of Ionic Strength of Dispersed Phase on Ostwald Ripening in Water-in-Oil Emulsions. Colloid J. 2003, 65, 40–43. 10.1023/A:1022362807131.

[ref71] Zhu Q.; Wu F.; Saito M.; Tatsumi E.; Yin L. Effect of magnesium salt concentration in water-in-oil emulsions on the physical properties and microstructure of tofu. Food Chem. 2016, 201, 197–204. 10.1016/j.foodchem.2016.01.065. 26868566

[ref72] Balmer J. A.; Armes S. P.; Fowler P. W.; Tarnai T.; Gáspár Z.; Murray K. A.; Williams N. S. J. Packing Efficiency of Small Silica Particles on Large Latex Particles: A Facile Route to Colloidal Nanocomposites. Langmuir 2009, 25, 5339–5347. 10.1021/la8041555. 19260684

[ref73] Fielding L. A.; Mykhaylyk O. O.; Armes S. P.; Fowler P. W.; Mittal V.; Fitzpatrick S. Correcting for a Density Distribution: Particle Size Analysis of Core–Shell Nanocomposite Particles Using Disk Centrifuge Photosedimentometry. Langmuir 2012, 28, 2536–2544. 10.1021/la204841n. 22214311

[ref74] Hoyt L. F. New Table of the Refractive Index of Pure Glycerol at 20°C. Ind. Eng. Chem. 1934, 26, 329–332. 10.1021/ie50291a023.

[ref75] Thormählen I.; Straub J.; Grigull U. Refractive Index of Water and Its Dependence on Wavelength, Temperature, and Density. J. Phys. Chem. Ref. Data 1985, 14, 933–945. 10.1063/1.555743.

[ref76] Katritzky A. R.; Sild S.; Karelson M. Correlation and Prediction of the Refractive Indices of Polymers by QSPR. J. Chem. Inf. Comput. Sci. 1998, 38, 1171–1176. 10.1021/ci980087w.

[ref77] György C.; Verity C.; Neal T. J.; Rymaruk M. J.; Cornel E. J.; Smith T.; Growney D. J.; Armes S. P. RAFT Dispersion Polymerization of Methyl Methacrylate in Mineral Oil: High Glass Transition Temperature of the Core-Forming Block Constrains the Evolution of Copolymer Morphology. Macromolecules 2021, 54, 9496–9509. 10.1021/acs.macromol.1c01528.

