# Supplementary material for: Sterically Stabilized Diblock Copolymer Nanoparticles Enable Efficient Preparation of Non-Aqueous Pickering Nanoemulsions
Source: Langmuir. 2023 May 15;39(21):7361–70. doi: 10.1021/acs.langmuir.3c00464 (PMC10233796; doi:10.1021/acs.langmuir.3c00464)
Supplement: Supplementary file 1 — la3c00464_si_001.pdf [file la3c00464_si_001.pdf]

Supporting Information for:

Sterically-stabilized Diblock Copolymer Nanoparticles Enable Efficient Preparation of Non-aqueous Pickering Nanoemulsions

Saul J. Hunter and Steven P. Armes*

Dainton Building, Department of Chemistry, Brook Hill,

University of Sheffield, Sheffield, South Yorkshire, S3 7HF, UK.

* Author to whom correspondence should be addressed (s.p.armes@shef.ac.uk)

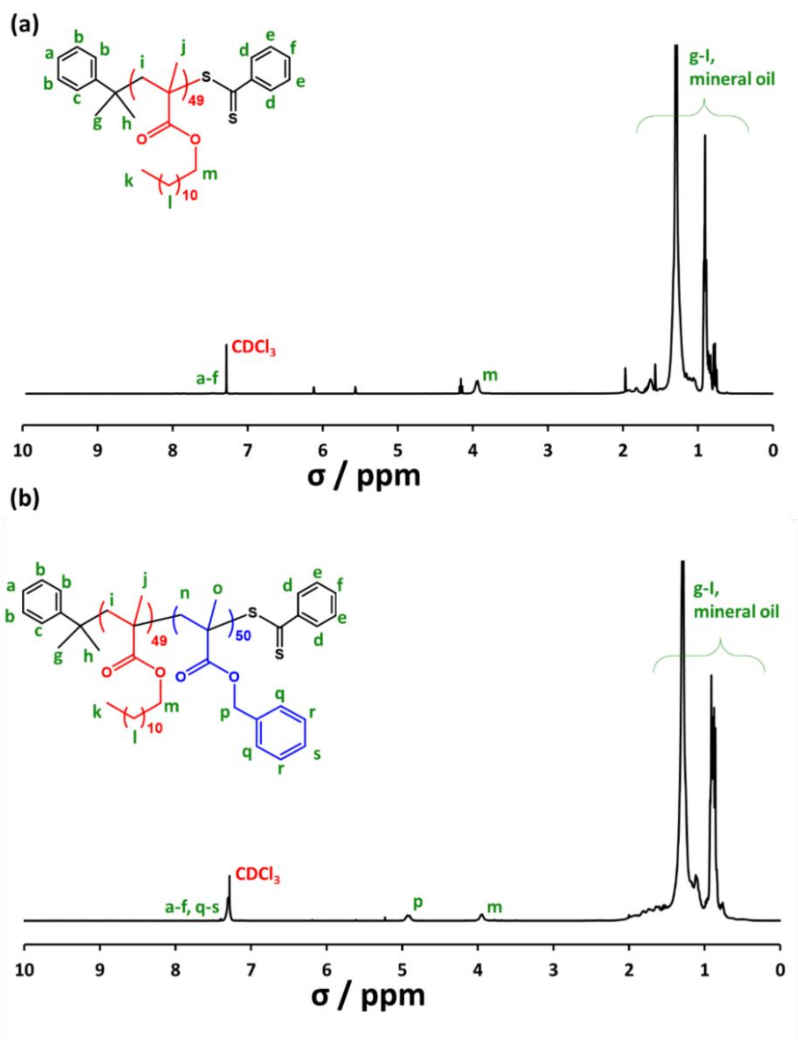

Figure S1. Assigned ^{1}H NMR spectra recorded in $CDCl_3$ for (a) the $PLMA_{49}$ precursor (LMA conversion = 97%) and (b) the $PLMA_{49}$-$PBzMA_{50}$ diblock copolymer (BzMA conversion = 99%) prepared using a one-pot PISA protocol at 20% w/w solids in mineral oil at 90 °C.

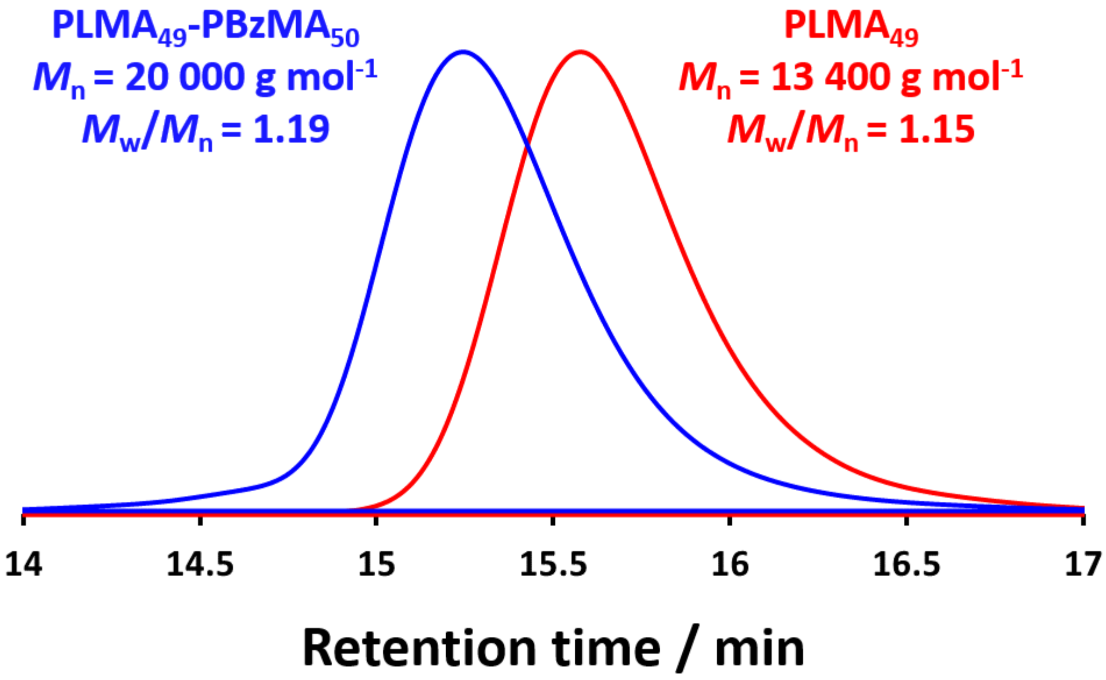

Figure S2. Overlaid THF GPC curves recorded for a $PLMA_{49}$ precursor and the corresponding $PLMA_{49}$-$PBzMA_{50}$ diblock copolymer. The M_n and M_w/M_n data are expressed relative to a series of near-monodisperse poly(methyl methacrylate) calibration standards.

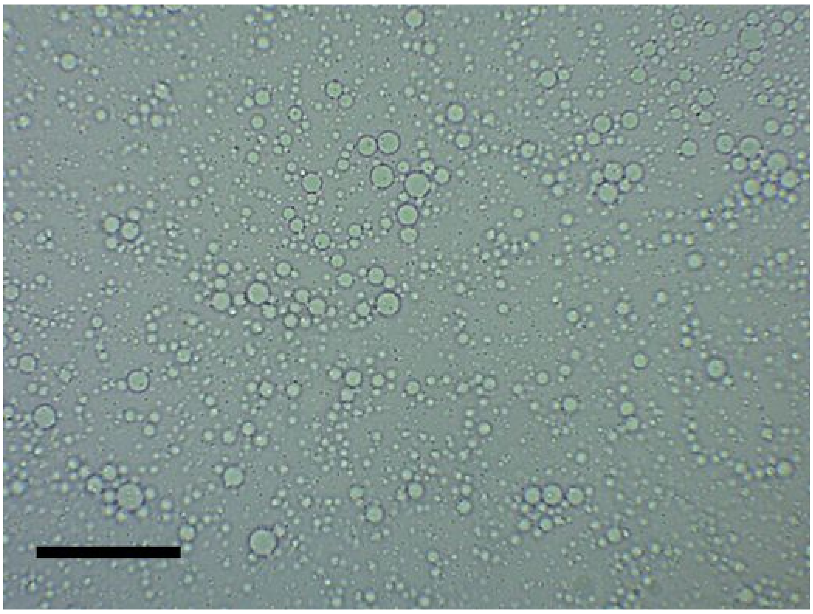

Figure S3. Optical microscopy image recorded for a non-aqueous glycerol-in-mineral oil Pickering macroemulsion prepared using a 5.0% w/w $PLMA_{49}$-$PBzMA_{50}$ nanoparticles in mineral oil. Emulsification conditions: high-shear homogenization (13 500 rpm, 2 min, 20 °C); glycerol volume fraction = 0.20. Scale bar = 30 µm.

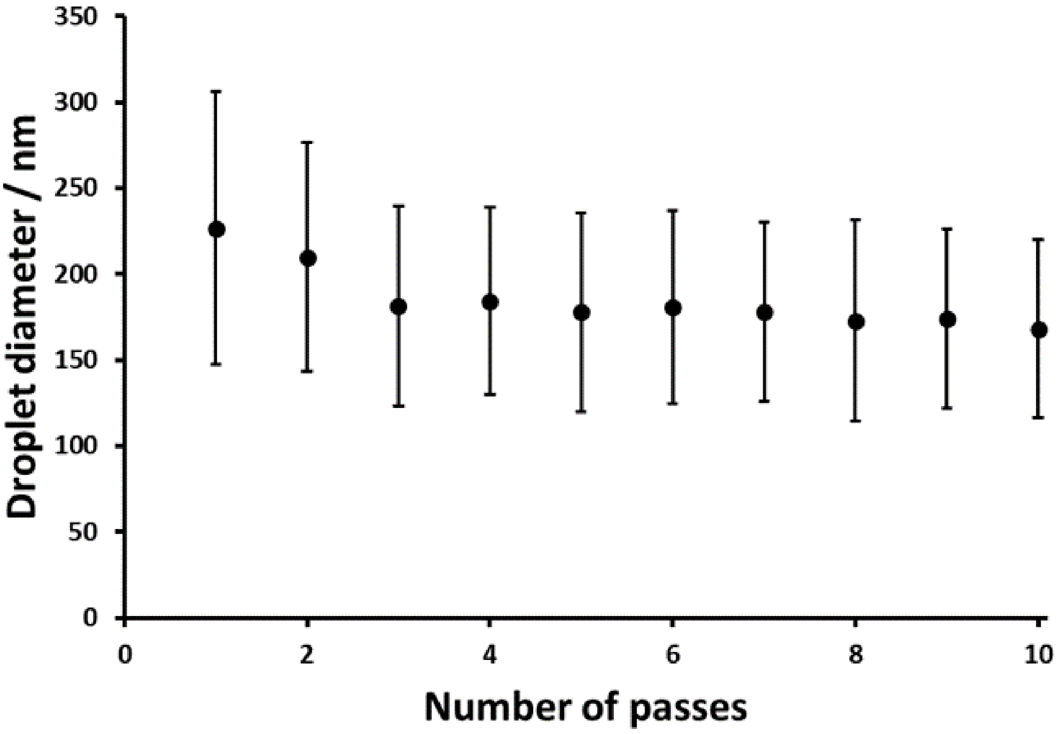

Figure S4. Variation in z-average droplet diameter vs. number of passes through an LV1 microfluidizer observed for a series of glycerol-in-mineral oil Pickering nanoemulsions prepared using 5.0% w/w $PLMA_{49}$-$PBzMA_{50}$ nanoparticles in mineral oil. Emulsification conditions: applied pressure = 20 000 psi; glycerol volume fraction = 0.20.

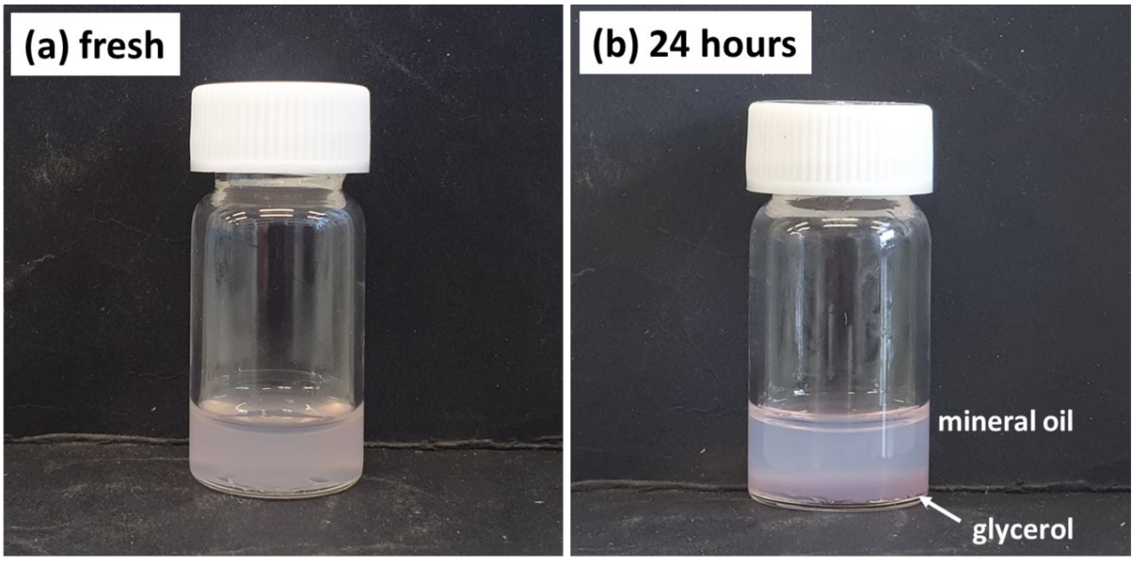

Figure S5. Digital photographs recorded for (a) a freshly prepared glycerol-in-mineral oil Pickering nanoemulsion and (b) the same nanoemulsion after aging for 24 h at 20 °C. Ostwald ripening causes rapid growth of the relatively dense glycerol droplets, which undergo gravitational sedimentation over time. Microfluidization conditions: applied pressure = 20 000 psi; 1 pass; glycerol volume fraction = 0.20.

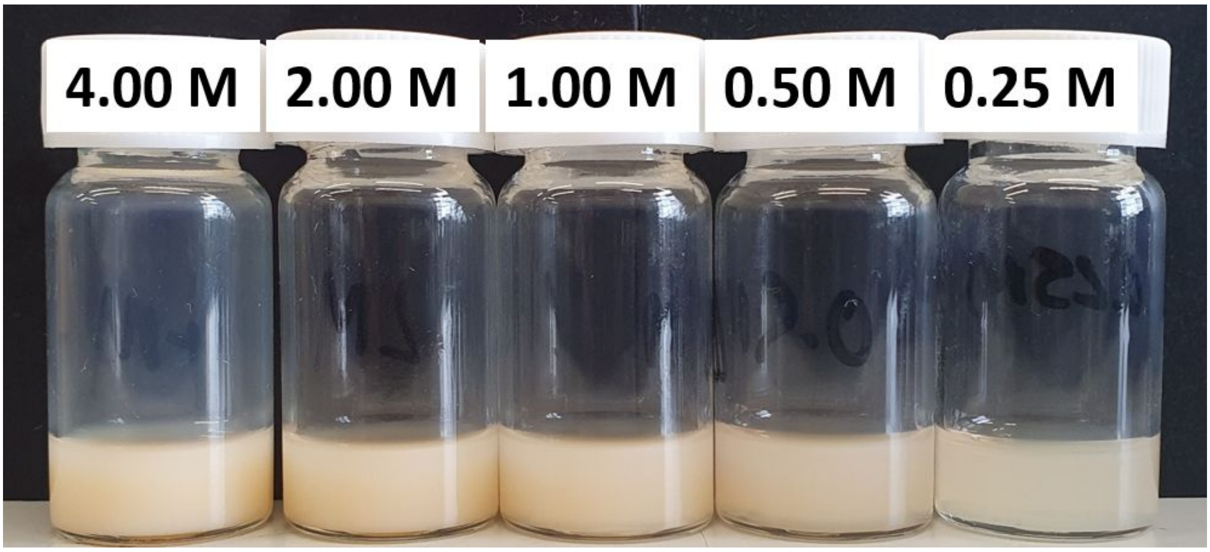

Figure S6. Digital photographs recorded for glycerol-in-mineral oil Pickering nanoemulsions prepared using 5.0% w/w $PLMA_{49}$-$PBzMA_{50}$ nanoparticles in mineral oil with 0.25 – 4.00 M NaI dissolved in the glycerol phase. Microfluidization conditions: applied pressure = 20 000 psi; 1 pass; glycerol volume fraction = 0.20.

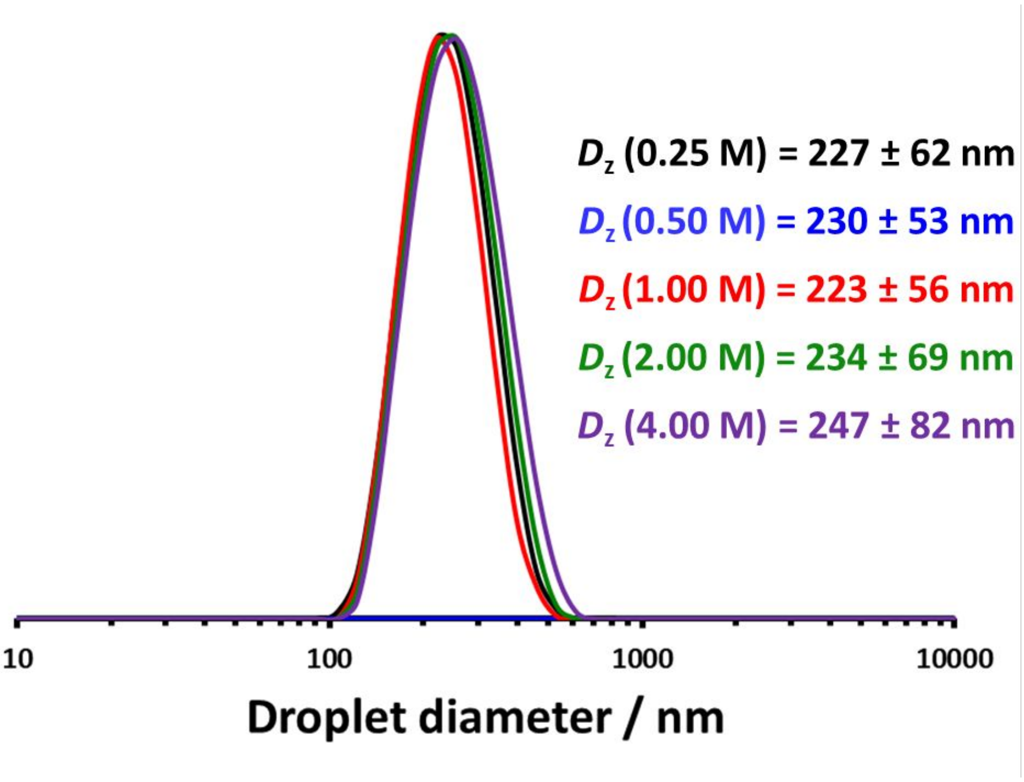

Figure S7. Overlaid intensity-average droplet size distributions determined using DLS for glycerol-in-mineral oil Pickering nanoemulsions prepared using 5.0% w/w $PLMA_{49}$-$PBzMA_{50}$ nanoparticles in mineral oil with 0.25 – 4.00 M NaI dissolved in the glycerol phase. Microfluidization conditions: applied pressure = 20 000 psi; 1 pass; glycerol volume fraction = 0.20.

Table S1. Summary of droplet density, droplet diameter, mean number of nanoparticles per droplet, and packing efficiency data determined for a series of glycerol-in-mineral oil Pickering nanoemulsions prepared using 5.0% w/w $PLMA_{49}$-$PBzMA_{50}$ nanoparticles in mineral oil with 0.00–4.00 M NaI dissolved within the glycerol phase. Microfluidization conditions: applied pressure = 20 000 psi; 1 pass; glycerol volume fraction = 0.20.

[NaI] (M)	Glycerol droplet density (g cm^{-3})	Initial DLS droplet diameter (nm)	Number of nanoparticles adsorbed per droplet (*N*)	Packing Efficiency, *P* (%)
0.00	1.00	231 ± 87	300	30
0.25	1.33	227 ± 62	271	29
0.50	1.47	230 ± 53	295	30
1.00	1.60	223 ± 56	267	29
2.00	1.88	234 ± 69	314	31
4.00	2.44	246 ± 82	376	33

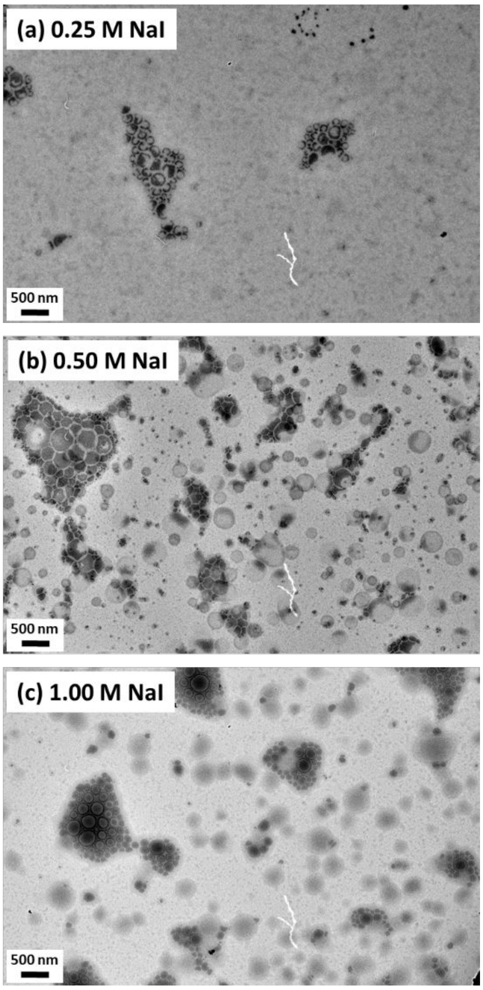

Figure S8. Representative TEM images recorded for dried glycerol-in-mineral oil nanoemulsions prepared using 5.0% w/w $PLMA_{49}$-$PBzMA_{50}$ nanoparticles in mineral oil with 0.25 – 1.00 M NaI dissolved in the glycerol phase. The NaI acts as a convenient heavy atom stain to improve the electron contrast. Conditions: applied pressure = 20 000 psi; 1 pass; volume fraction of (glycerol + NaI) droplet phase = 0.20.

SAXS Model

The spherical micelle SAXS model[1] employed in this study has been reported in detail elsewhere.[2] The scattering length densities used for the coronal PLMA (ξ_{PLMA}) and core PBzMA (ξ_{PBzMA}) blocks were 8.82 x 10^{10} cm^{-2} and 10.38 x 10^{10} cm^{-2}, respectively, and that for the mineral oil solvent (ξsol) was 7.63 x 10^{10} cm^{-2}. Programming tools within the Irena SAS Igor Pro macros[3] were used to implement the scattering model.

References

1. Pedersen, J., Form factors of block copolymer micelles with spherical, ellipsoidal and cylindrical cores. *J. Appl. Crystallogr.* **2000,** *33*, 637-640.

2. Cornel, E. J.; Smith, G. N.; Rogers, S. E.; Hallett, J. E.; Growney, D. J.; Smith, T.; O'Hora, P. S.; van Meurs, S.; Mykhaylyk, O. O.; Armes, S. P., Time-resolved small-angle neutron scattering studies of the thermally-induced exchange of copolymer chains between spherical diblock copolymer nanoparticles prepared via polymerization-induced self-assembly. *Soft Matter* **2020,** *16*, 3657-3668.

3. Ilavsky, J.; Jemian, P. R., Irena: tool suite for modeling and analysis of small-angle scattering. *Journal of Applied Crystallography* **2009,** *42*, 347-353.
